# Supplementary material for: Characterization of the horse chestnut genome reveals the evolution of aescin and aesculin biosynthesis
Source: Nat Commun. 2023 Oct 13;14:6470. doi: 10.1038/s41467-023-42253-y (PMC10576086; doi:10.1038/s41467-023-42253-y)
Supplement: Supplementary file 1 — Supplementary information [file 41467_2023_42253_MOESM1_ESM.pdf]

Characterization of the horse chestnut genome reveals the evolution of aescin and aesculin biosynthesis

Sun *et al.*

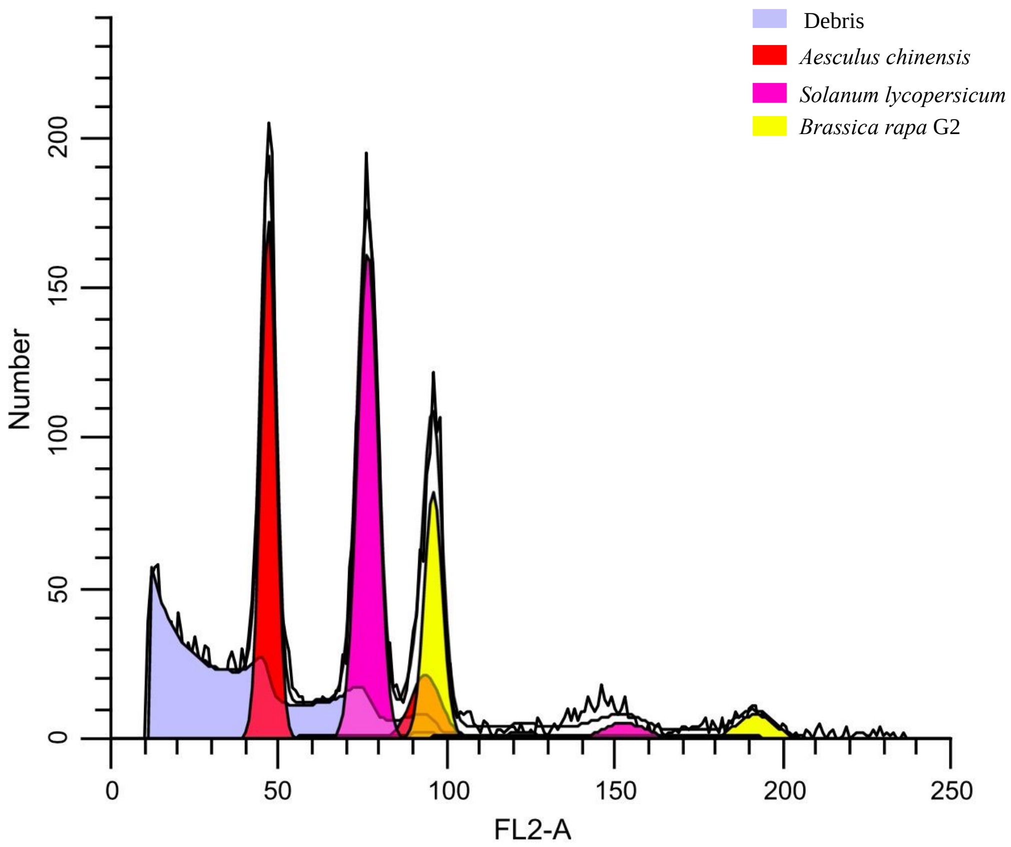

Supplementary Figure 1. Flow cytometry is used to estimate the genome size of *A. chinensis* using *Solanum lycopersicum* (900 Mb) and *Brassica rapa* (485 Mb) as the standards. The yellow peak at FL2-A value of 47.10 for *Brassica rapa* was covered by the peak of *A. chinensis* (46.82). The genome size was estimated as 481.9 Mb. Source data are provided as a Source Data file.

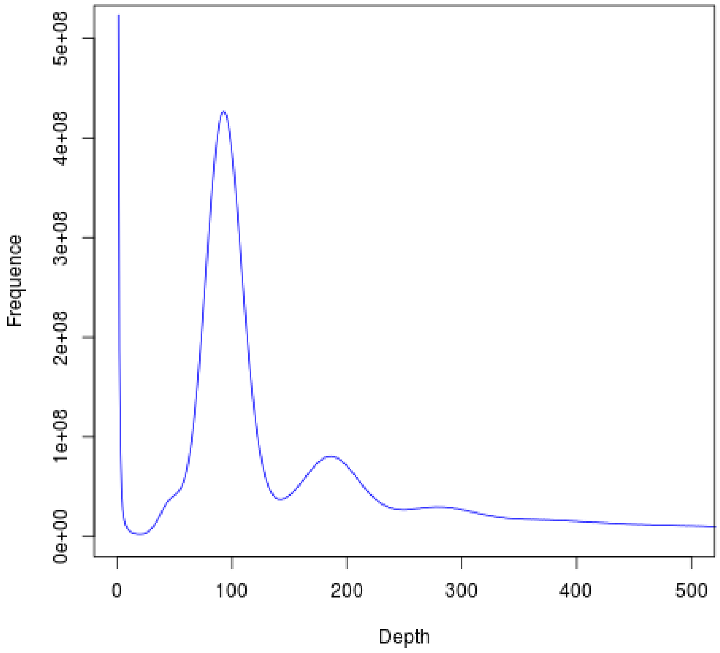

Supplementary Figure 2. The genome size of *A. chinensis* estimated by 17 *k*-mer. We totally identified 47,364,609,904 *K*-mers and the peak depth of *K*-mer is 92. The genome size of *A. chinensis* was calculated as 514.83 Mb, and the revised genome size is 504.38 Mb.

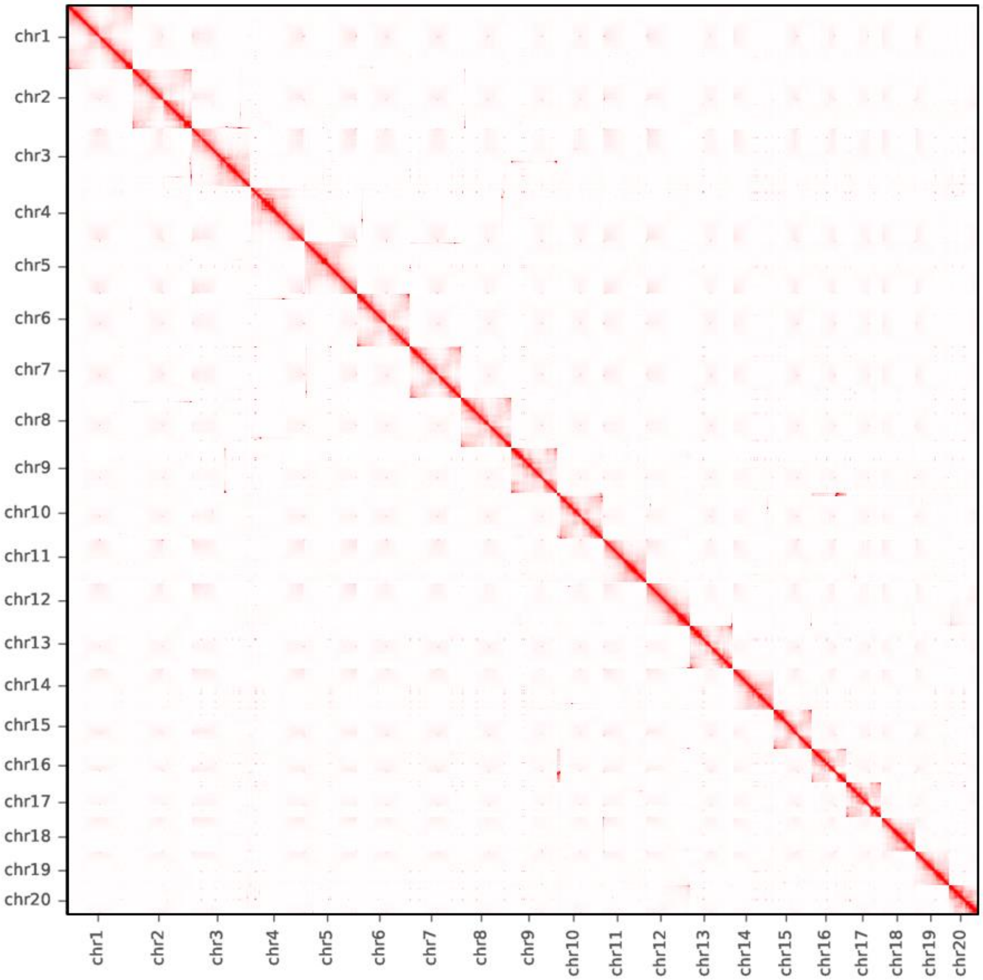

Supplementary Figure 3. Heatmap of *A. chinensis* chromosome conformation capture analysis.

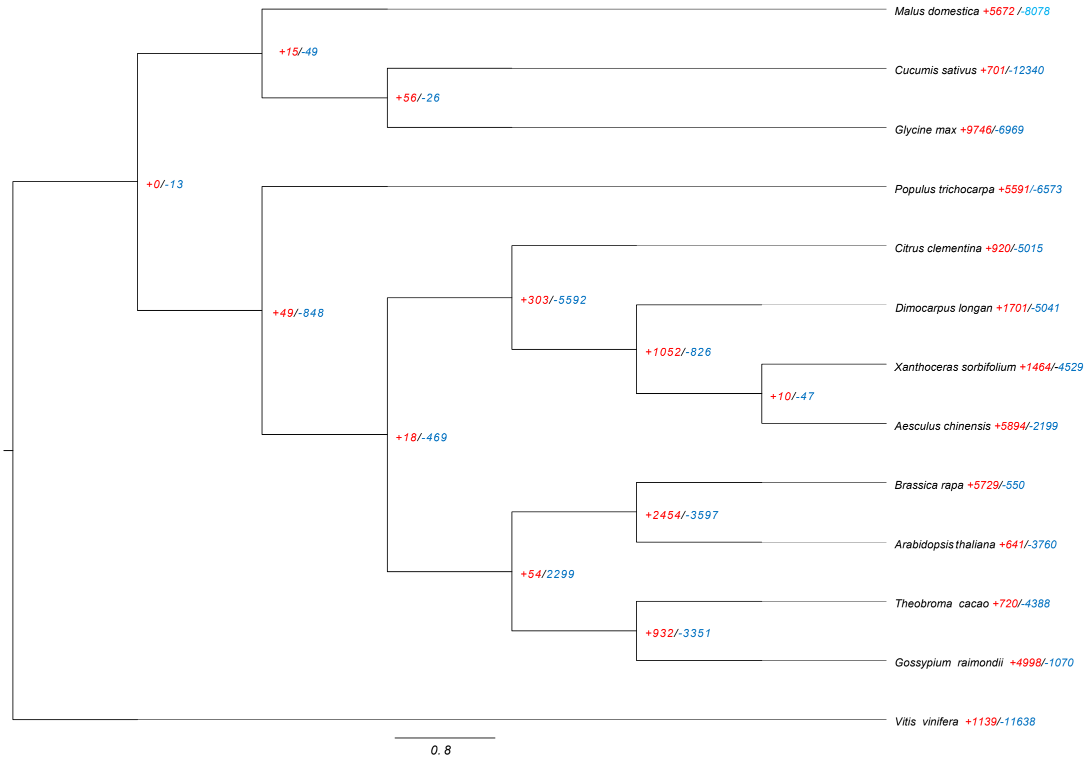

Supplementary Figure 4. The orthologous genes and phylogenetic analysis among *A. chinensis* and other 12 angiosperms. The red and blue numbers present the expansion and contraction of gene families among 13 angiosperms respectively.

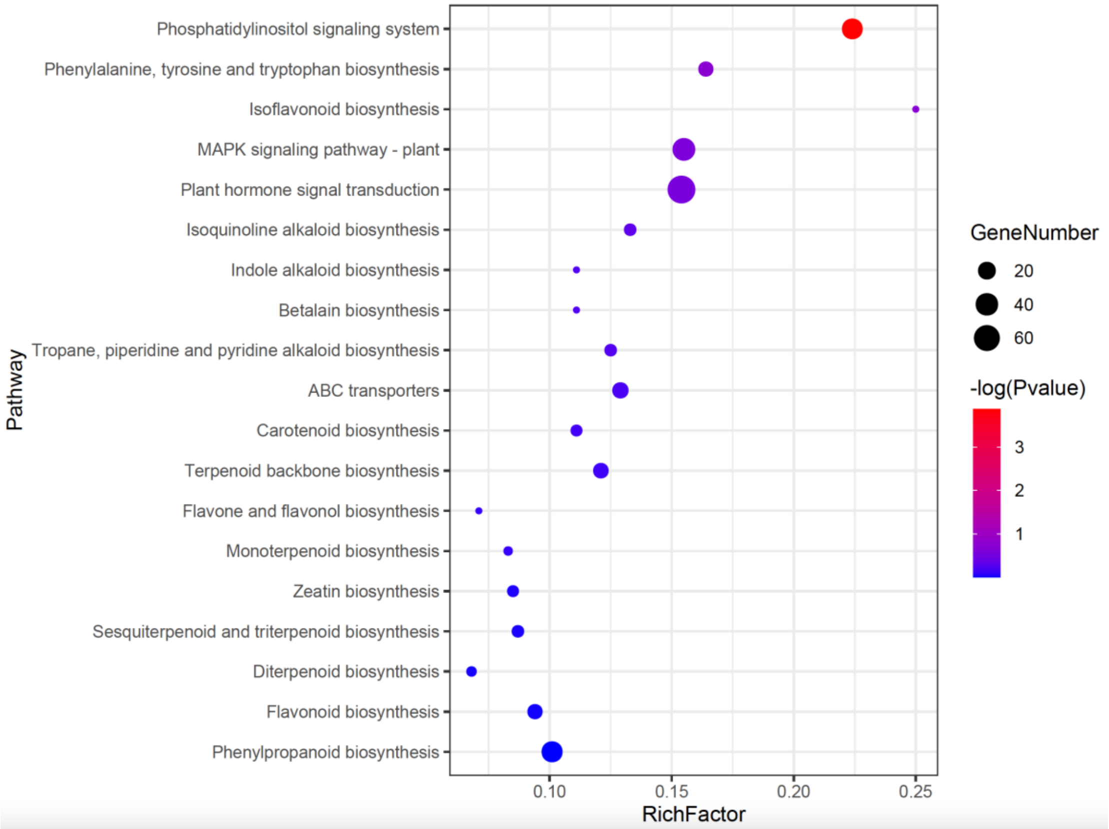

Supplementary Figure 5. KEGG pathway enrichment analysis for the expanded gene families in *A. chinensis* genome. The size of each circle means the amounts of genes. The different color of each circle means the *P*-value. RichFactor means the number of genes in the enriched KEGG pathway divided by the number of background genes. Source data are provided as a Source Data file.

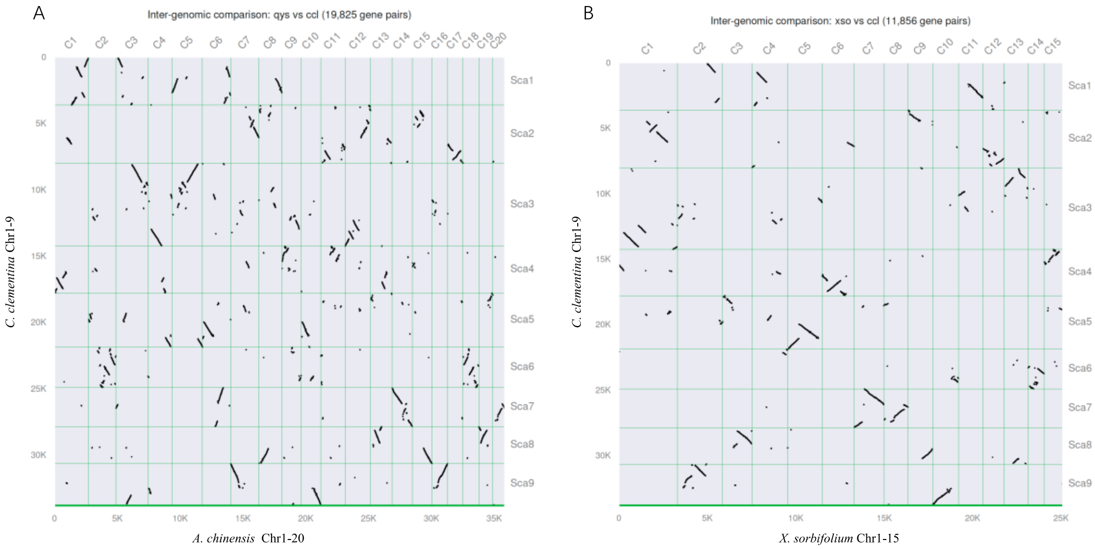

Supplementary Figure 6. Synteny analysis among the *A. chinensis*, *C. clementina*, and *X. sorbifolium* genomes. Dot plots showed the syntenic orthologs between *A. chinensis* and *C. clementina* (A), and between *C. clementina*, and *X. sorbifolium* (B).

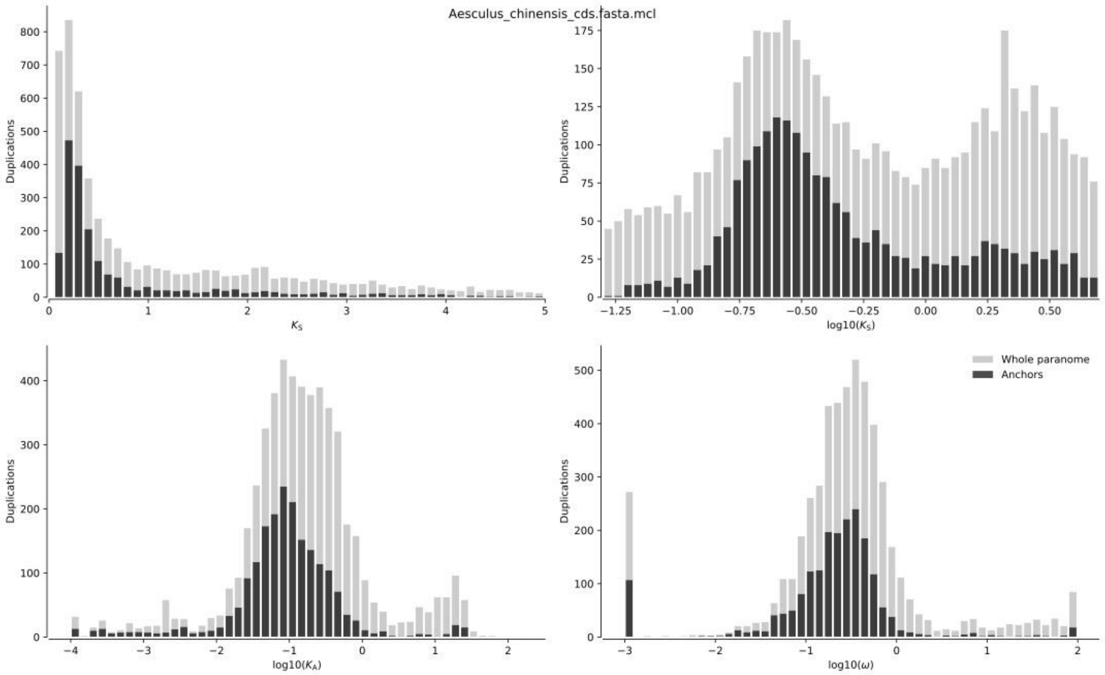

Supplementary Figure 7. K_S distributions of whole paralogs (grey bars) and anchor-pair paralogs (black bars) for *A. chinensis*. Two obvious peaks in the $\log_{10}$ (K_S) distributions indicated that there are at least two WGD events that occurred during the evolution of *A. chinensis*.

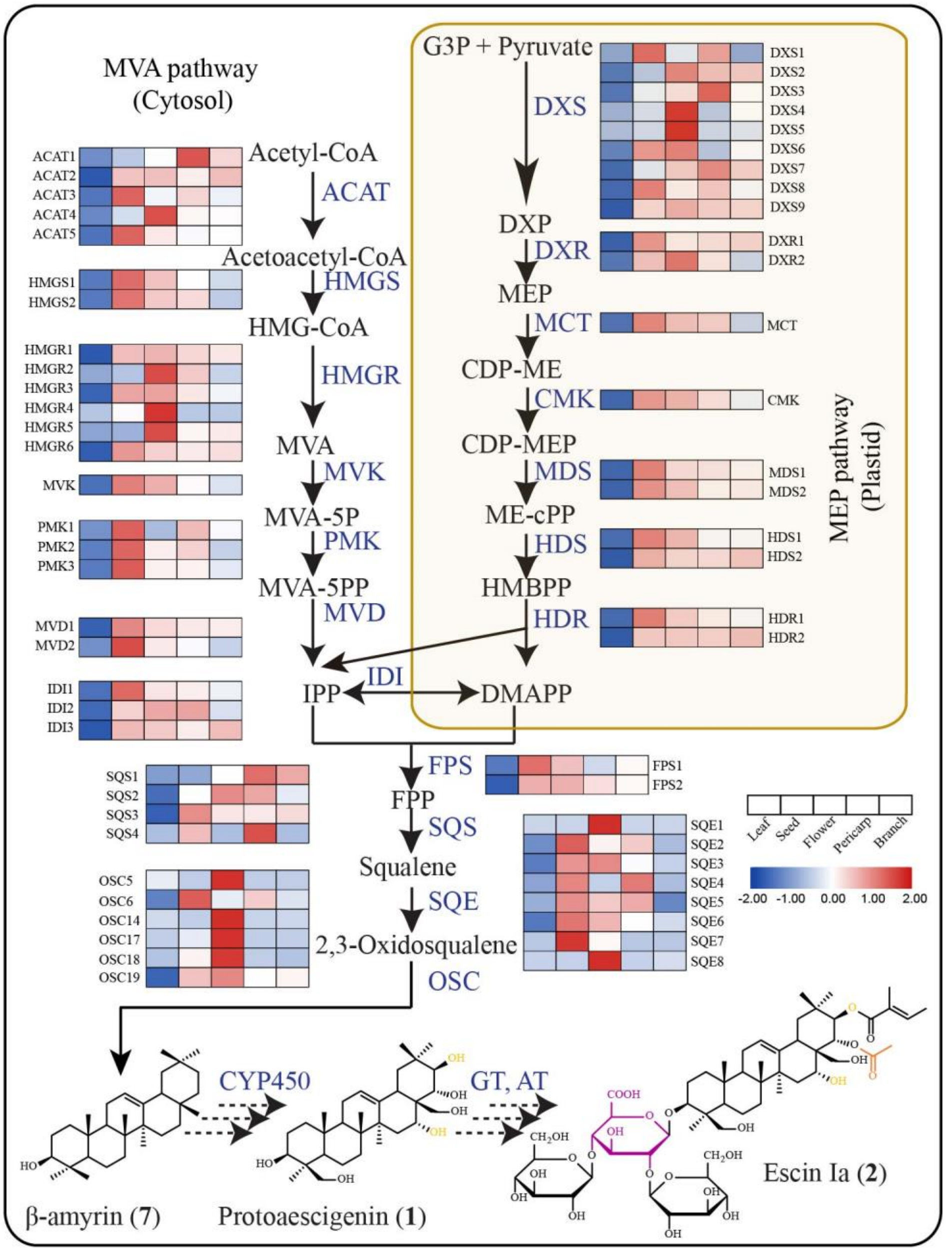

Supplementary Figure 8. The gene expression heatmap of MEP and MVA genes contribute to biosynthesis of triterpenoids in *A. chinensis*. 1: Protoaescigenin; **2**: Escin Ia; **7**: β-amyrin. Source data are provided as a Source Data file.

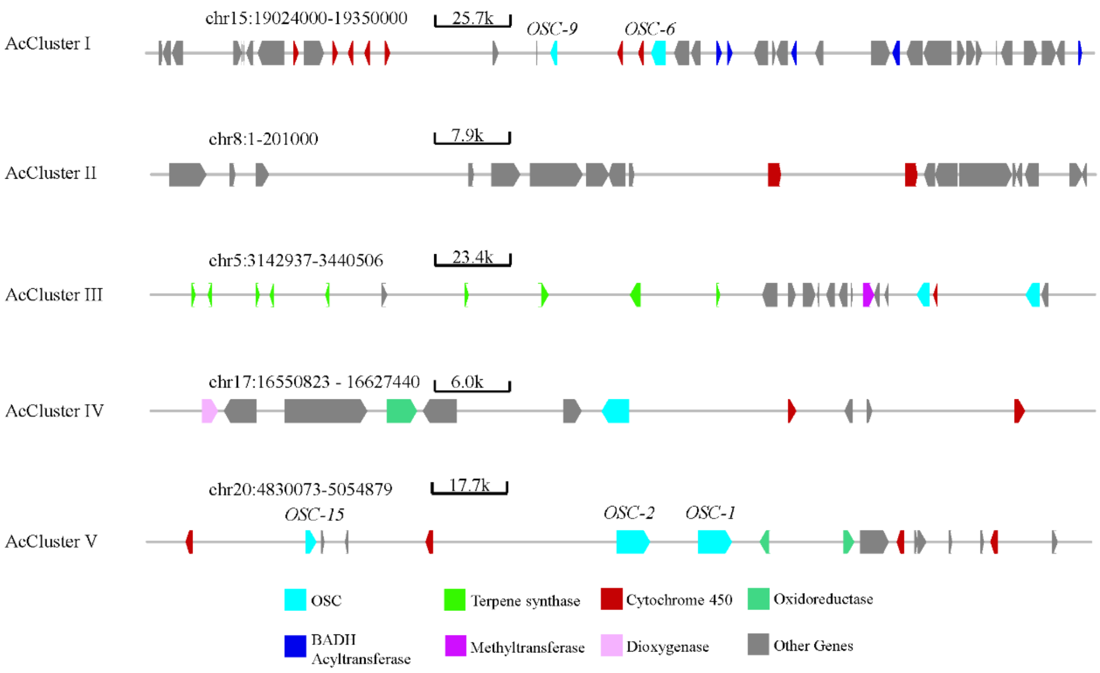

Supplementary Figure 9. Five biosynthetic gene clusters (AcClusters I-V) related to triterpenoid biosynthesis in *A. chinensis* genome. AcClusters I, III, IV and V containing skeleton OSCs and tailoring genes were searched in each chromosome of *A. chinensis* genome via the online tools of plantiSMASH (http://plantismash.secondarymetabolites.org/). AcCluster II was manually found.

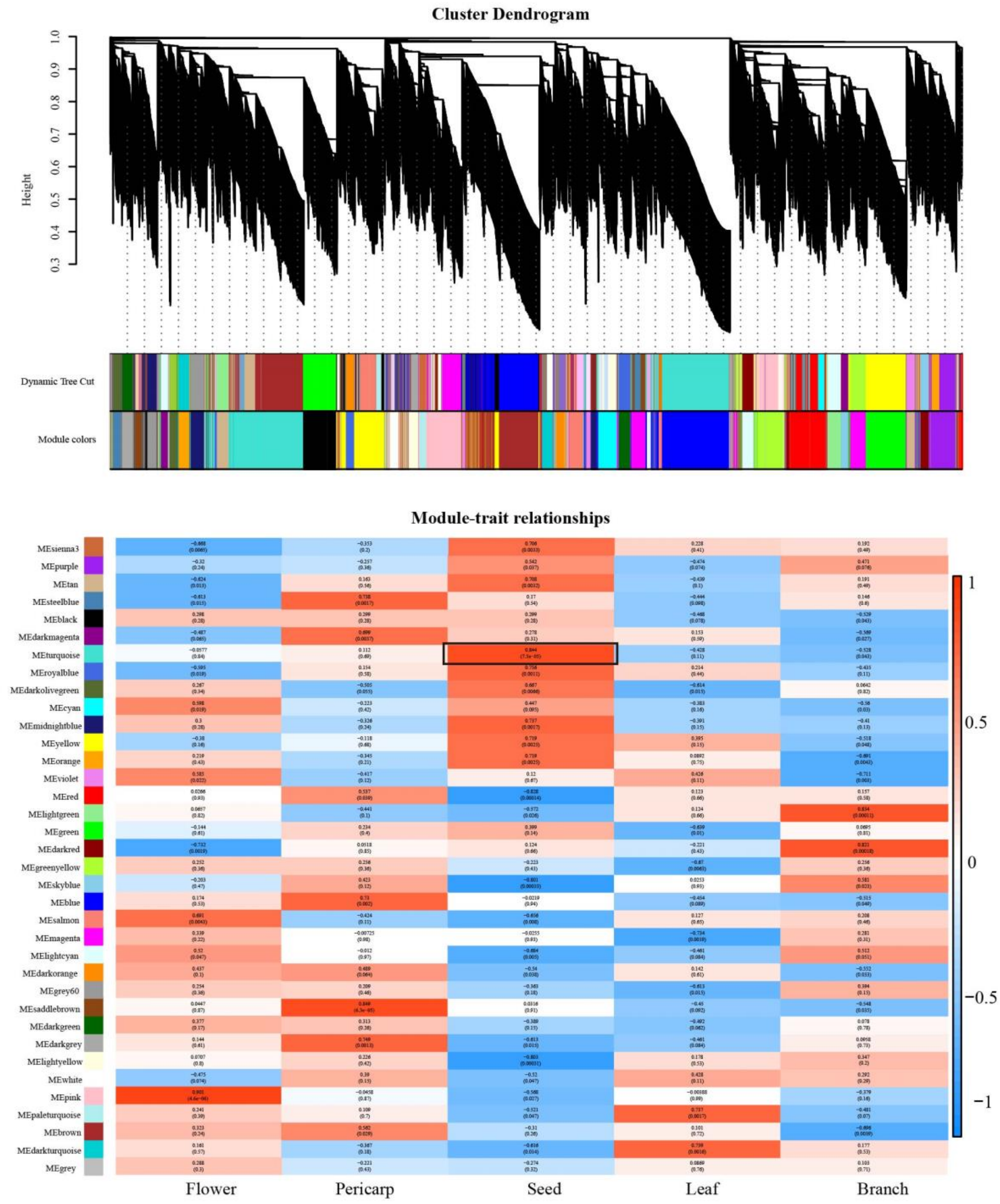

Supplementary Figure 10. The WGCNA "turquoise" module associated with aescin biosynthesis. Represented as a baits AcOSC6 and AcCLS1 connecting candidate P450, BAHDs and UGTs, see Supplementary Data 6.

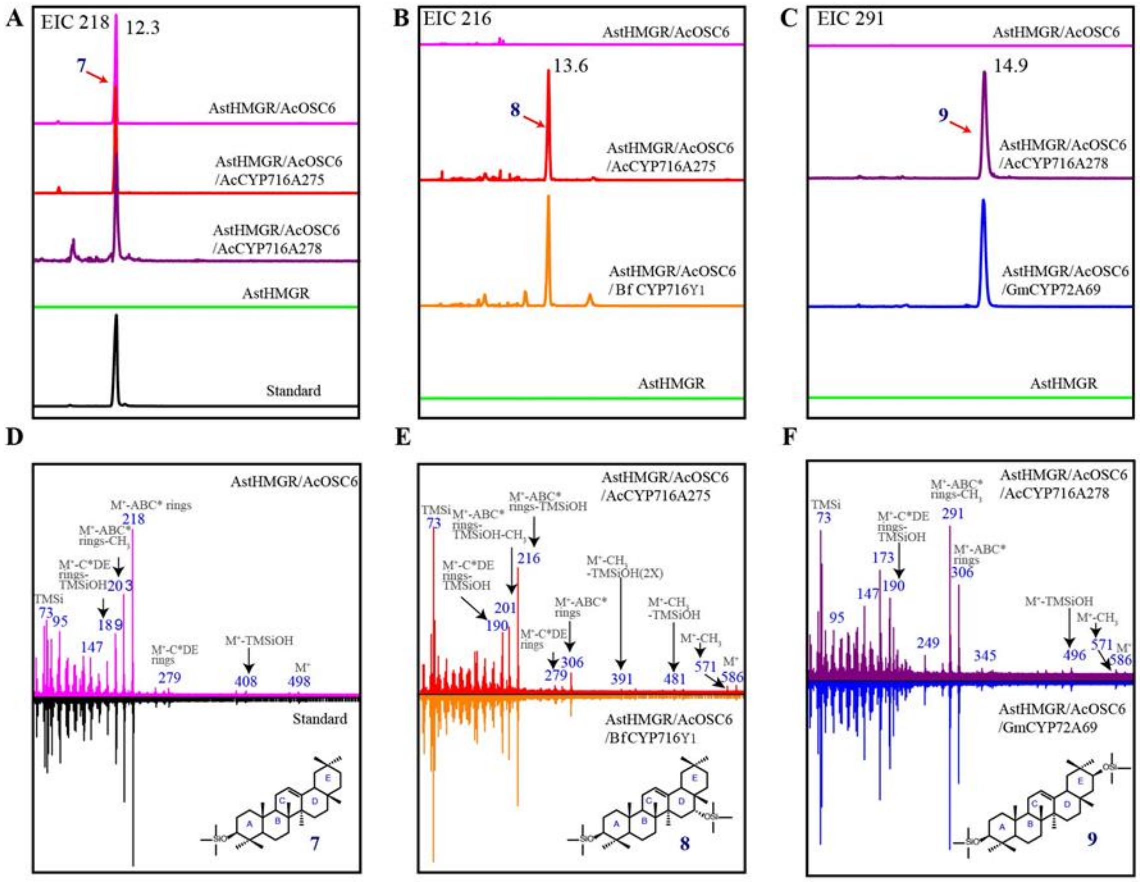

Supplementary Figure 11. The function identification of AcOSC6, AcCYP716A275 and AcCYP716A278 through the *N. benthamiana* platform. (A) EIC of β-amyrin (*m/z* 218; positive ion mode) of samples from *N. benthamiana* leaves transiently expressing AstHMGR combined with AcOSC6, and with AcCYP716A275 or AcCYP716A278 compared to plants expressing AstHMGR alone and to β-amyrin standard. (B) EIC of 16α-hydroxy-β-amyrin (*m/z* 216; positive ion mode) of products from *N. benthamiana* leaves transiently expressing AstHMGR combined with AcOSC6, and with AcCYP716A275 or BfCYP716Y1. (C) EIC of 21β-hydroxy-β-amyrin (*m/z* 291; positive ion mode) of products from *N. benthamiana* leaves transiently expressing AstHMGR combined with AcOSC6, and with AcCYP716A278 or GmCYP72A69. (D, E, F) GC-MS results of β-amyrin, 16α-hydroxy-β-amyrin and 21β-hydroxy-β-amyrin. **7**: β-amyrin; **8**: 16α-hydroxy-β-amyrin; **9**: 21β-hydroxy-β-amyrin. Source data are provided as a Source Data file.

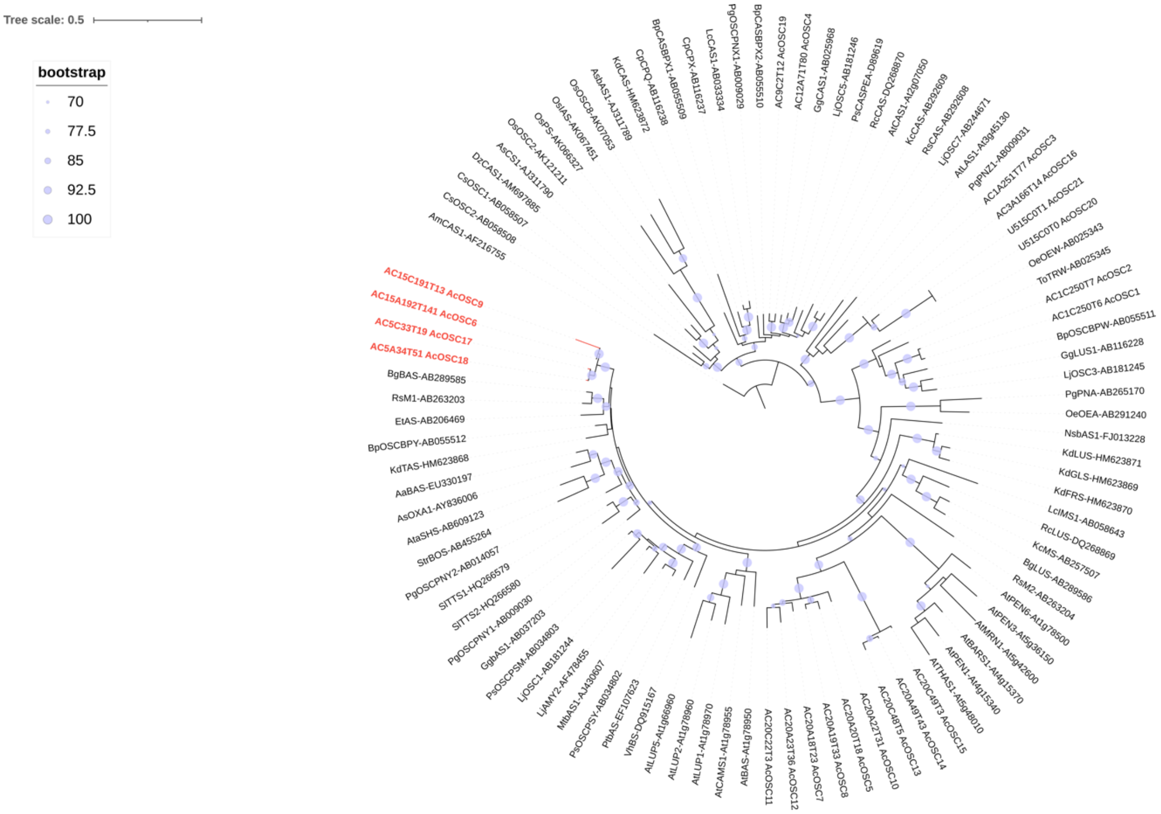

Supplementary Figure 12. Phylogenetic analysis of OSC genes annotated from *A. chinensis* genome with the representatives of characterized OSC genes from different species. The grey circles on the branch represented nodes with a support value greater than 70%. Source data are provided as a Source Data file.

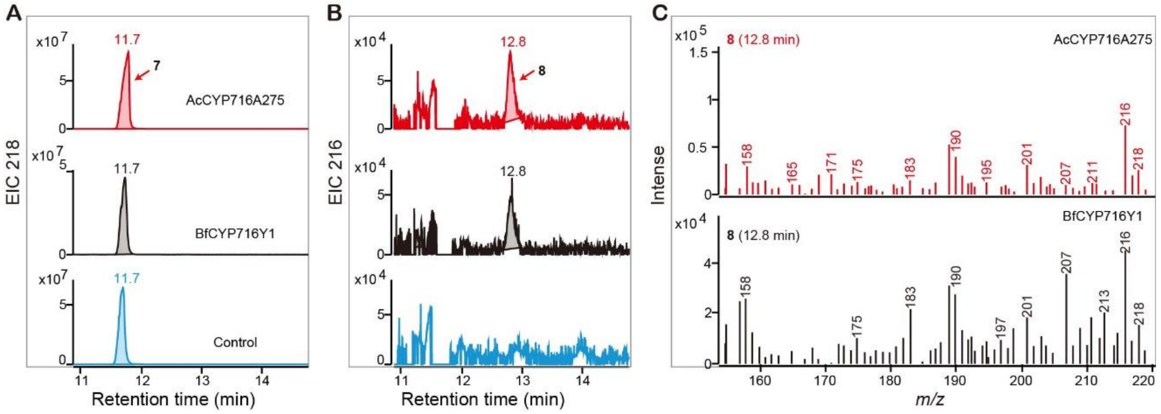

Supplementary Figure 13. Expression of AcCYP716A275 results in the accumulation of 16α-hydroxy-β-amyrin in engineered yeast strains. Retention time (A) and mass spectrum (B, C) to the products of BfCYP716Y1. **7**: β-amyrin; **8**: 16α-hydroxy-β-amyrin. Source data are provided as a Source Data file.

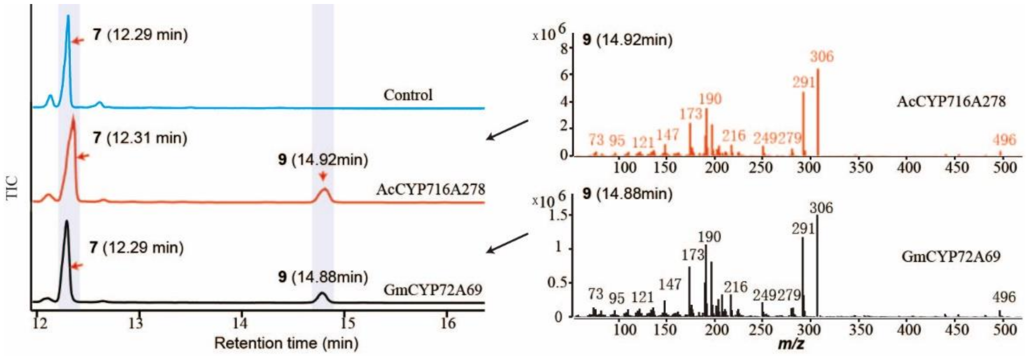

Supplementary Figure 14. Expression of AcCYP716A278 results in the accumulation of 21β-hydroxy-β-amyrin in engineered yeast strains. Retention time (left) and mass spectrum (right) to the products of GmCYP72A69. **7**: β-amyrin; **9**: 21β-hydroxy-β-amyrin. Source data are provided as a Source Data file.

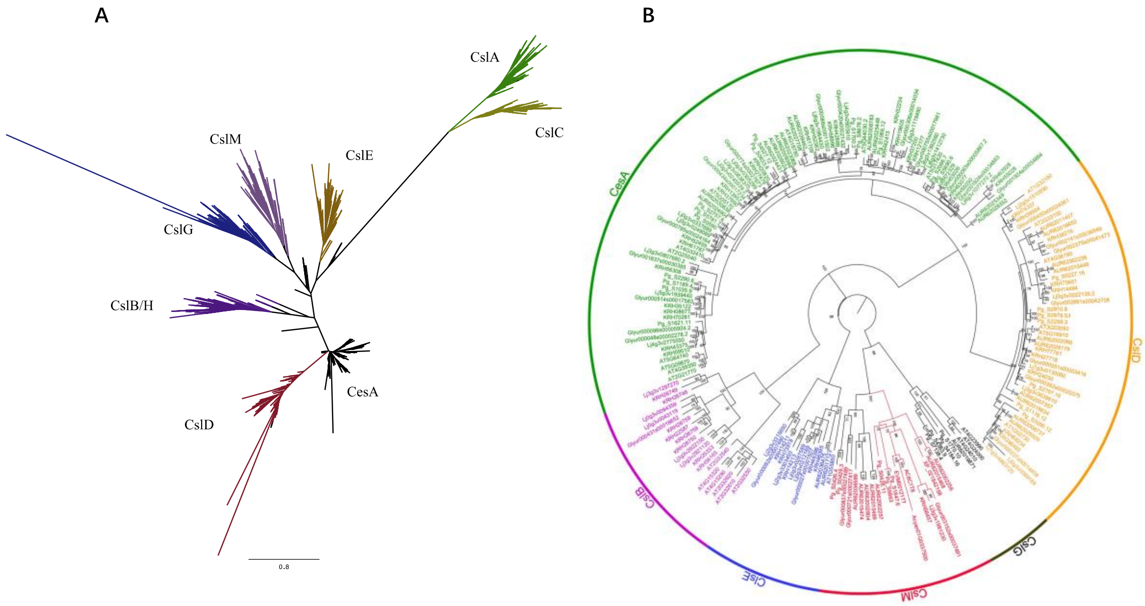

Supplementary Figure 15. Phylogenetic analysis of cellulose-synthase genes. (A) Phylogenetic analysis of 6268 cellulose-synthase superfamily genes from 144 reported angiosperm genomes. (B) The phylogenetic position of AcCSL with other proteins reported in the literature. The cellulose-synthase superfamily genes were annotated using PFAM of PF03552. Maximum likelihood tree was constructed using RAxML with 1000 bootstrap replicates. The branch support values greater than 70% were presented. Source data are provided as a Source Data file.

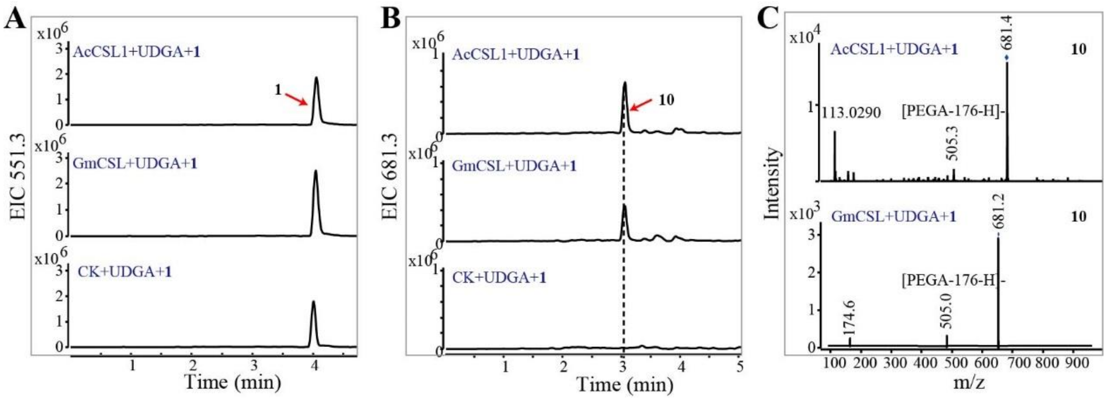

Supplementary Figure 16. Enzymatic assays of AcCSL1 and GmCSyGT-containing microsomes. (A) Overlay of LC-QQQ-MS/MS extracted ion chromatograms for protoaescigenin (*m/z* 551.3589) detected in microsomes expressing AcCSL1 and GmCSyGT1. (B) Overlay of LC-QQQ-MS/MS extracted ion chromatograms for 3-*O*-β-D-glucuronopyranosyl acid-protoaescigenin (*m/z* 681.3856) produced in microsomes expressing AcCSL1 and GmCSyGT. (C) MS spectrums of AcCSL1 and GmCSyGT1 catalytic products. **1**: Protoaescigenin; **10**: 3-O-β-D-glucuronopyranosyl acid-protoaescigenin. Source data are provided as a Source Data file.

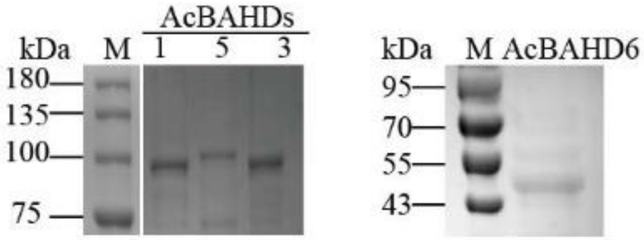

Supplementary Figure 17. SDS-PAGE of the purified AcBAHD proteins. M: Protein marker. Source data are provided as a Source Data file.

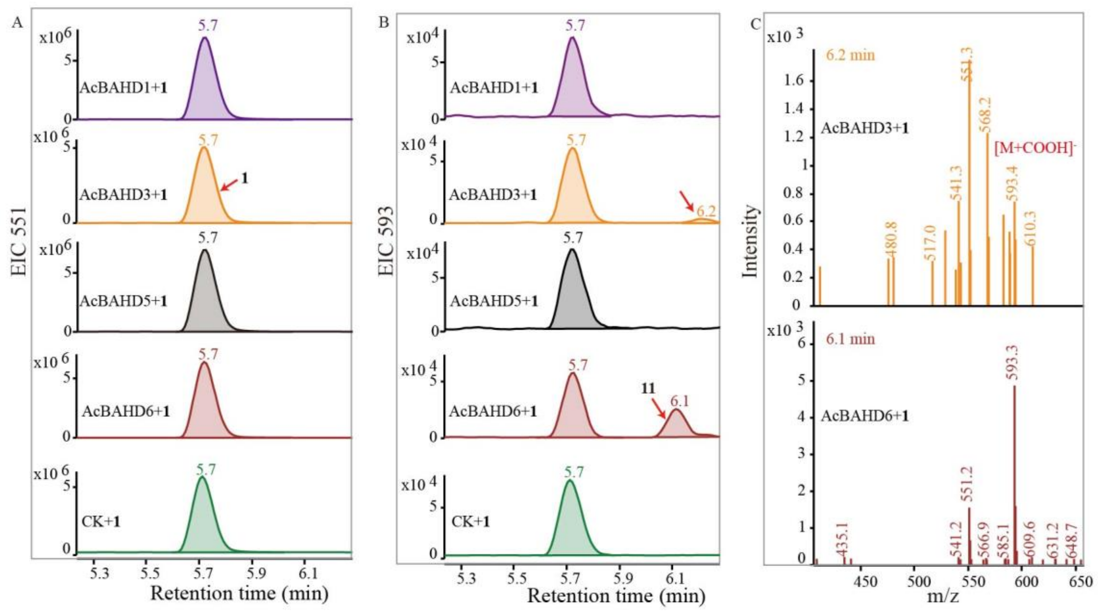

Supplementary Figure 18. Enzymatic assays of AcBAHD3 and AcBAHD6 with protoaescigenin being substrate. Overlay of LC-QQQ-MS/MS extracted ion chromatograms protoaescigenin (*m/z* 551) (A) and 22-*O*-acetylprotoaescigenin (*m/z* 593) (B) detected *in vitro* reactions expressing AcBAHD1, AcBAHD3, AcBAHD5 or AcBAHD6. (C) LC-QQQ-MS/MS spectrums of acylation products of protoaescigenin detected *in vitro* reactions expressing AcBAHD3 or AcBAHD6. **1**: Protoaescigenin; **11**: 22-O-acetylprotoaescigenin. Source data are provided as a Source Data file.

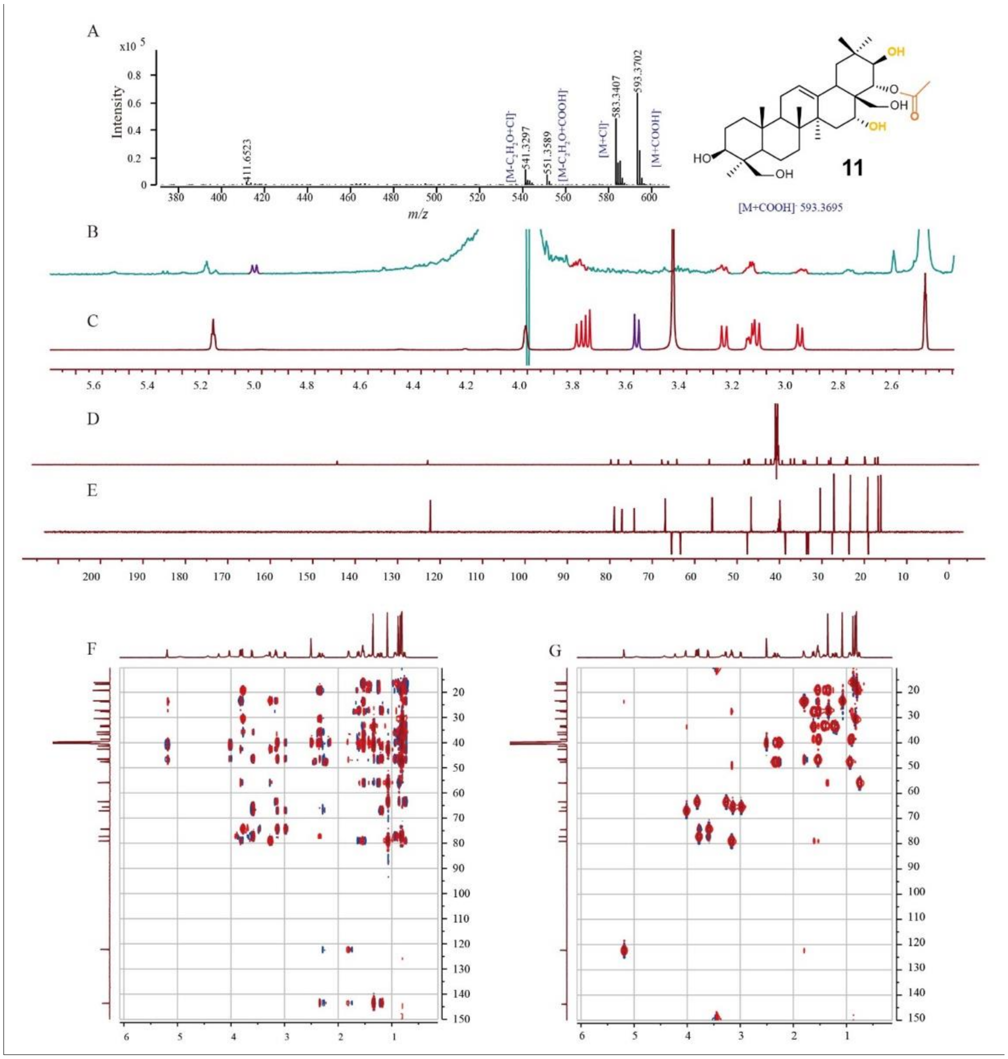

Supplementary Figure 19. LC-QTOF-MS/MS of spectrum 22-*O*-acetylprotoaescigenin (A) and NMR spectra of protoaescigenin and 22-*O*-acetylprotoaescigenin. (B) ^{1}H spectrum for 22-*O*-acetylprotoaescigenin in Dimethylsulfoxide-d6 (δ ppm). (B-E) NMR spectra of protoaescigenin in Dimethylsulfoxide-d6 (δ ppm): (C) ^{1}H; (D) ^{13}C; (E) DEPT-135; (F) DEPT-edited-HSQC; (G) HMBC. The results of ^{1}H-NMR showed that H_{β}-22 was at δ 3.59 in protoaescigenin, whereas in its acetate, the doublet was shifted to δ 5.01. A previous study reported that the ^{1}H-NMR of acetylation sites of protoaescigenin, including Hα-3 and Hα-21, generate a chemical shift of δ 1.28 and 1.47, respectively. Therefore, we speculate the acetate in this study was 22-*O*-acetylprotoaescigenin (Konoshima, Takao, and Kuo-Hsiung Lee. 1986. Antitumor Agents, 82. Cytotoxic Sapogenols from Aesculus hippocastanum. J NAT PROD 49 (4):650-6.). **11**: 22-O-acetylprotoaescigenin.

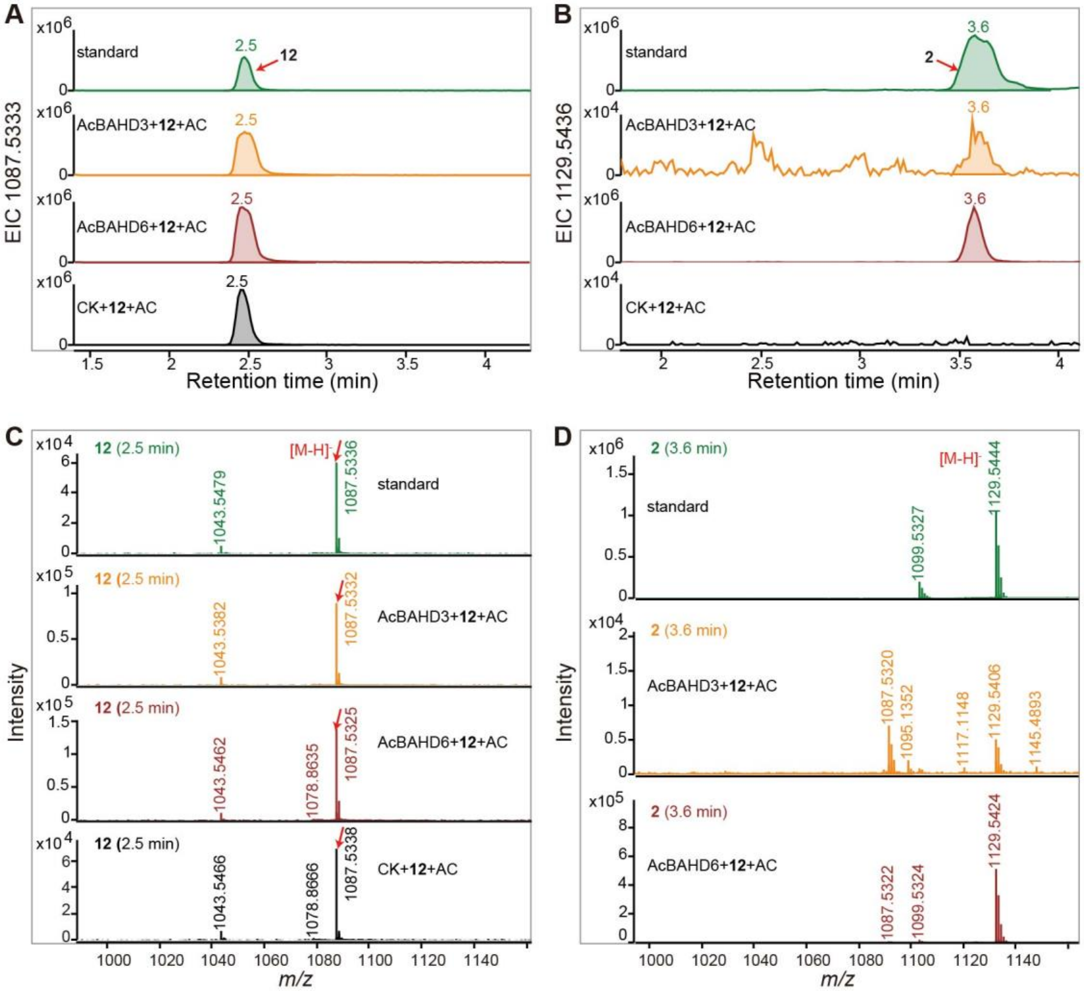

Supplementary Figure 20. Enzymatic assays of AcBAHD3 and AcBAHD6 with desacetylescin Ia being substrate. Overlay of LC-QTOF-MS/MS extracted ion chromatograms for desacetylescin Ia (*m/z* 1087.5333) (A) and escin Ia (*m/z* 1129.5436) (B) detected in corresponding standard or in vitro reactions expressing AcBAHD3 or AcBAHD6. MS spectrums of desacetylescin Ia (C) and escin Ia (D) were detected in corresponding standard or *in vitro* reactions expressing AcBAHD3 or AcBAHD6. **2**: Escin Ia; **12**: Desacetylescin Ia. Source data are provided as a Source Data file.

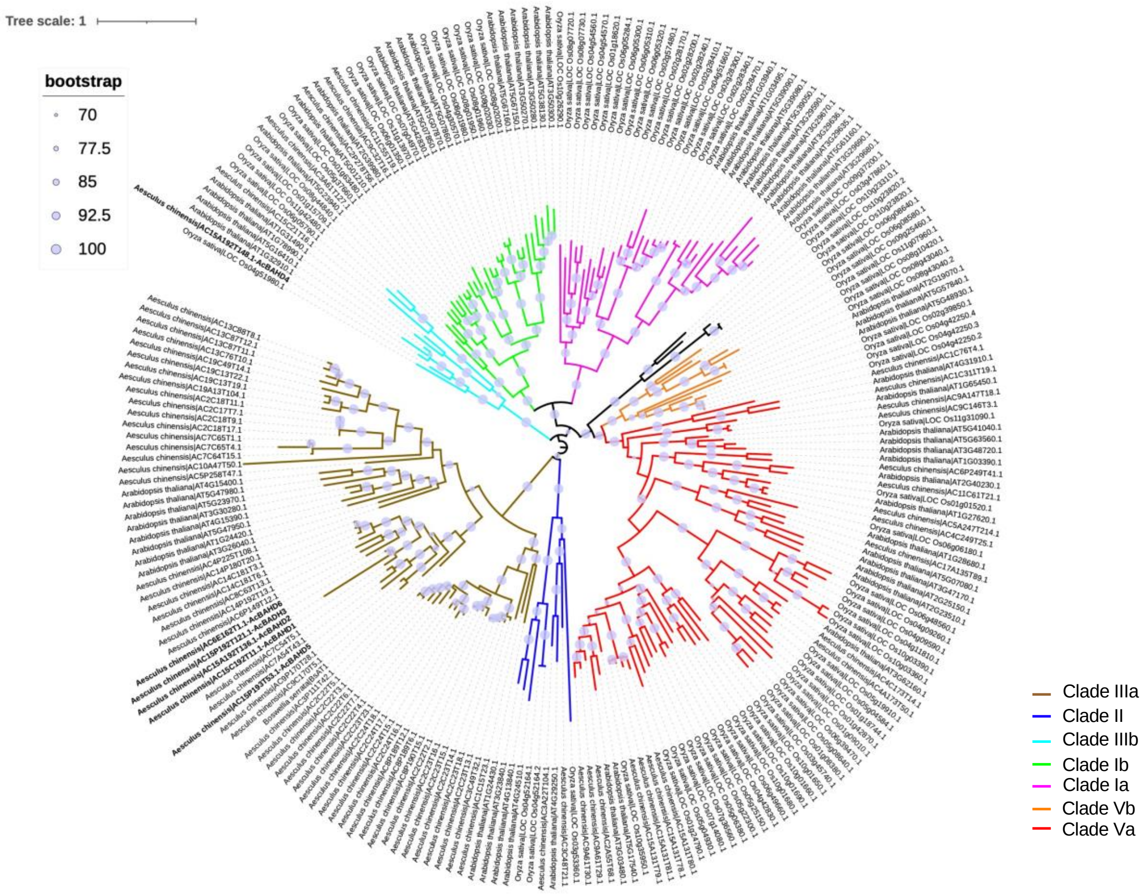

Supplementary Figure 21. Phylogenetic analysis of BAHD genes annotated from *A. chinensis* genome with the representatives of characterized BAHD genes from different species. The grey circles on the branch represented nodes with a support value greater than 70%. Source data are provided as a Source Data file.

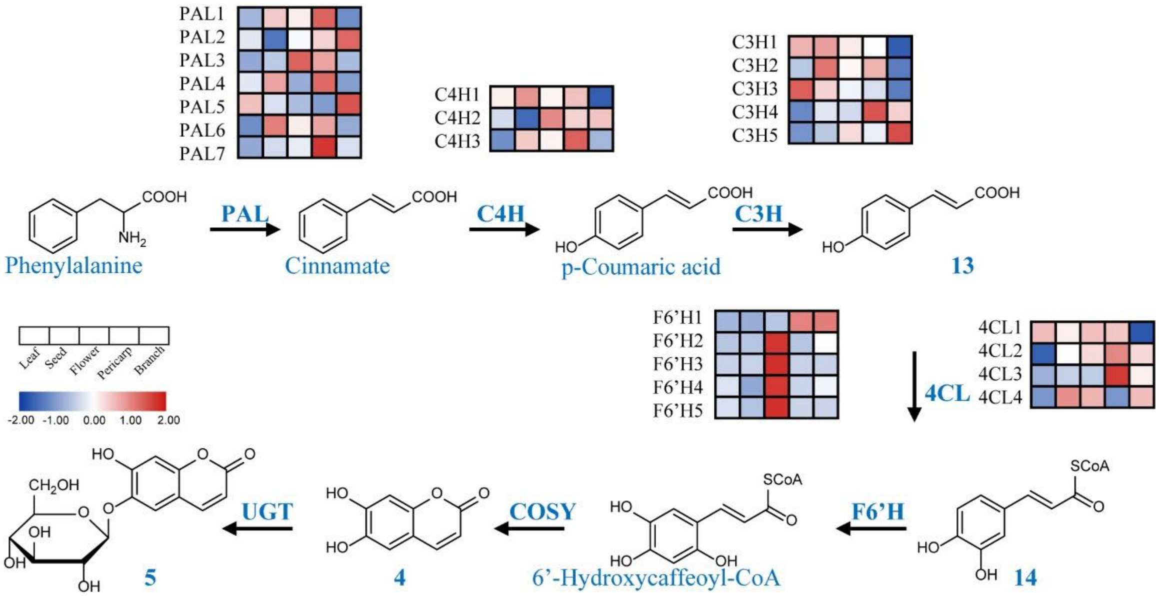

Supplementary Figure 22. Genes involved in aesculin biosynthesis. The biosynthesis of aesculin is represented in this simplified form. Genes associated with pathways identified by BLASTP. The expression value for each gene is indicated in color on $\log_{10}$ (FPKM + 1) scale for five tissues: leaf, seed, flower, pericarp, and branch. **4**: Esculetin; **5**: Aesculin; **13**: Caffeic acid; **14**: Caffeoyl-CoA. Source data are provided as a Source Data file.

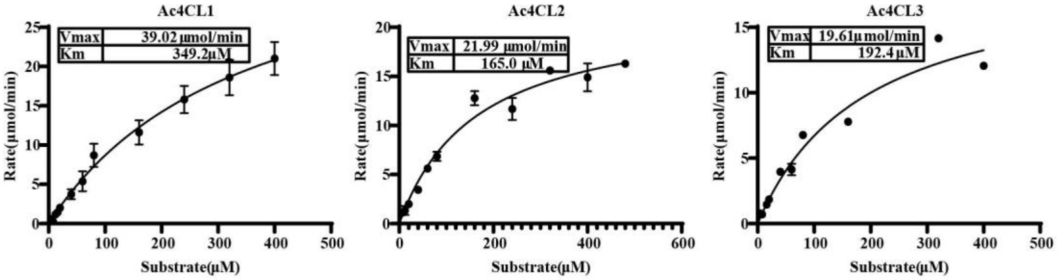

Supplementary Figure 23. Enzymatic kinetics of Ac4CL1, Ac4CL2, and Ac4CL3 with caffeic acid as substrate, respectively.

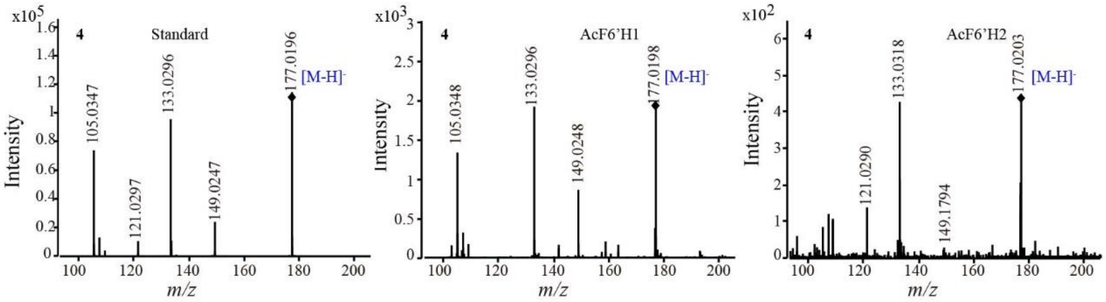

Supplementary Figure 24. LC-QTOF-MS/MS spectrums of esculetin standard and products of *in vitro* reactions expressing AcF6'H1 or AcF6'H2. 4: Esculetin. Source data are provided as a Source Data file.

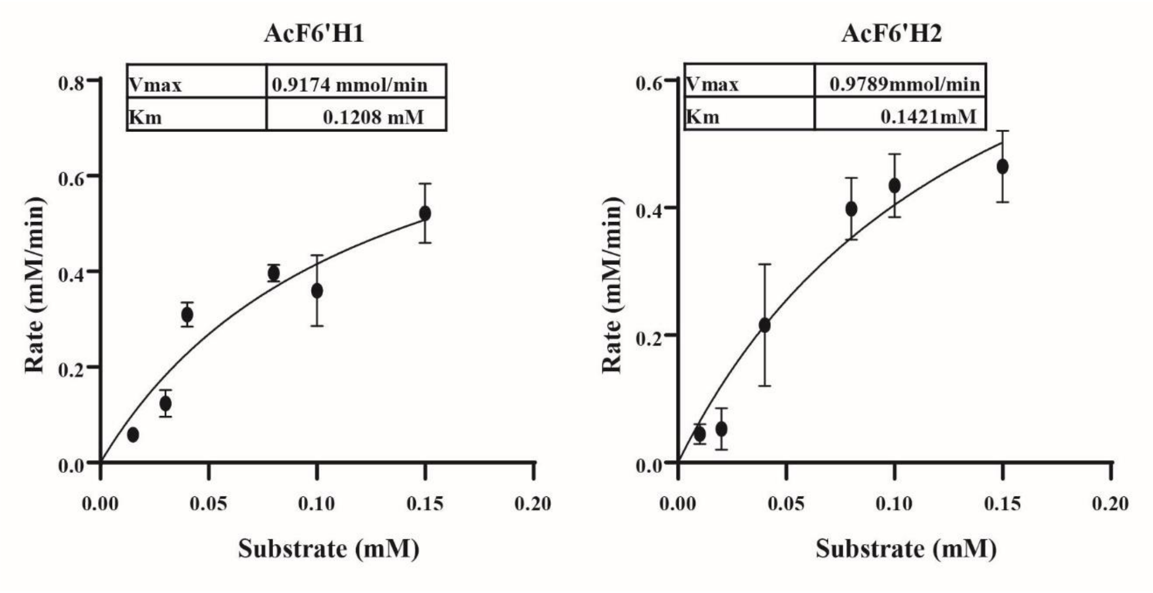

Supplementary Figure 25. Enzymatic kinetics of AcF6'H1 and AcF6'H2 with caffeoyl-CoA as substrate, respectively. Source data are provided as a Source Data file.

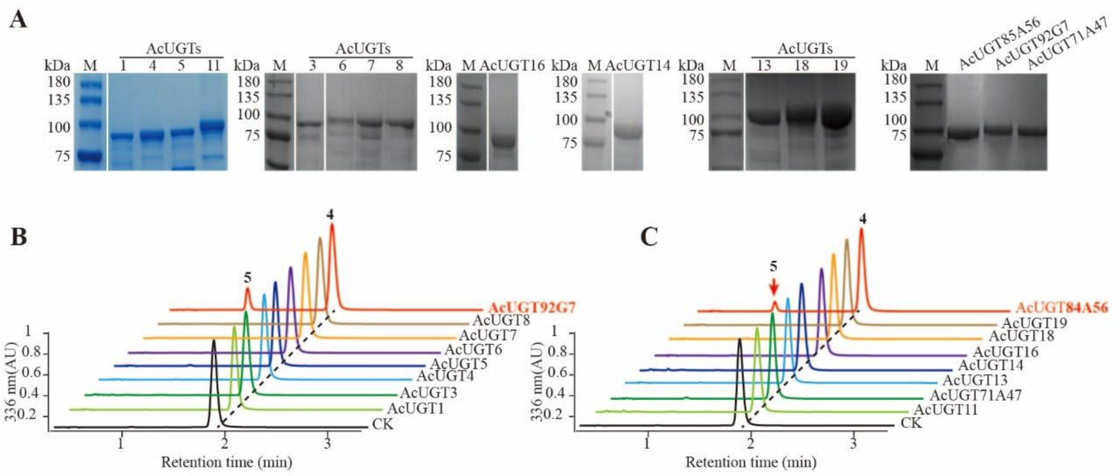

Supplementary Figure 26. SDS-PAGE of the AcUGT proteins (A), and the UPLC spectrums of their enzyme activity analysis (B, C). 4: Esculetin; **5**: Aesculin. Source data are provided as a Source Data file.

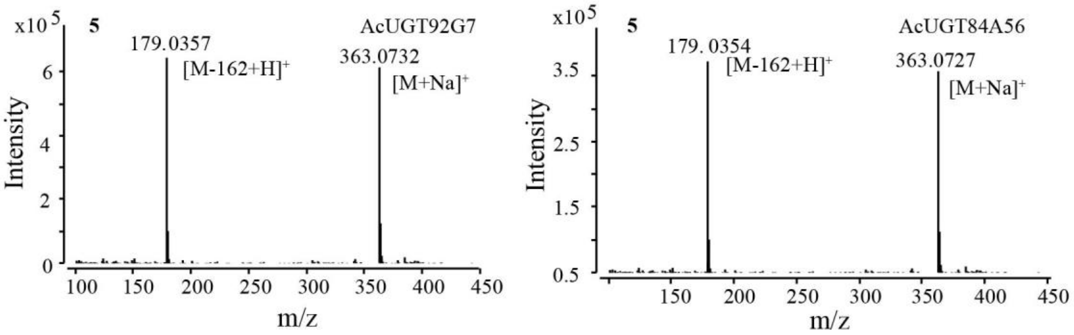

Supplementary Figure 27. LC-QTOF-MS/MS spectrums of aesculin produced by *in vitro* reactions expressing AcUGT92G7 or AcUGT84A56 towards esculetin. 5: Aesculin.

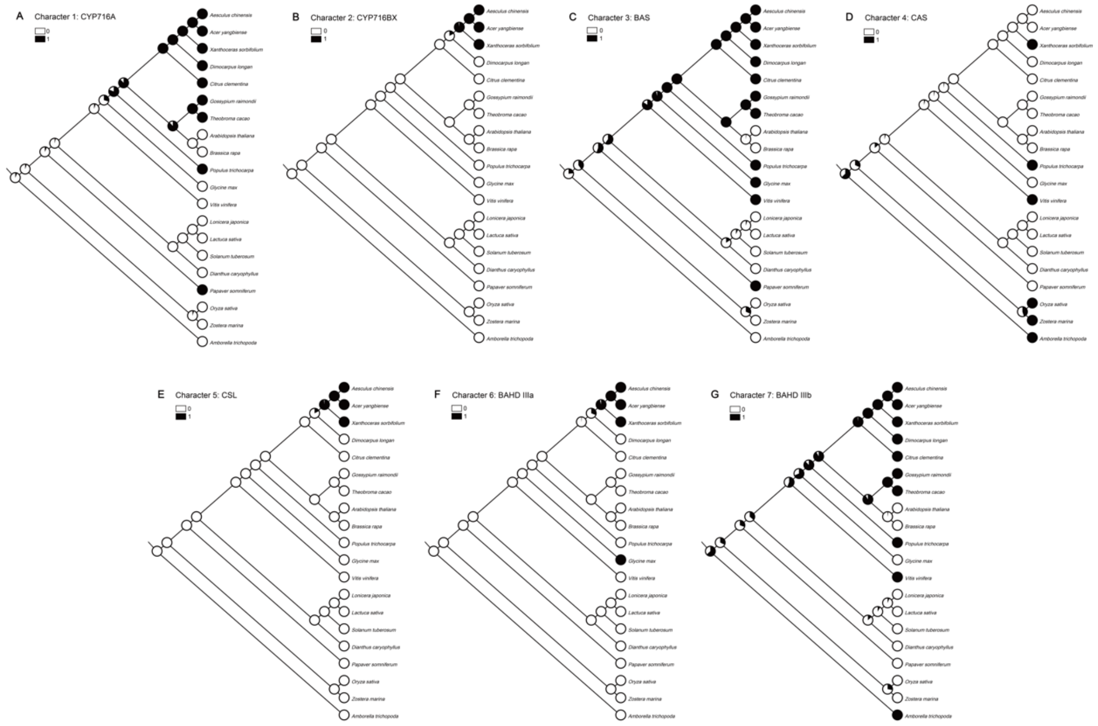

Supplementary Figure 28. Ancestral state reconstructions of barrigenol-type triterpenoid biosynthetic gene clusters among angiosperms. The binary traits were assigned to each leaf according to the presence (=1) or absence (=0) of targeted genes. A: CYP716A, B: CYP716BX, C: BAS, D: CAS, E: CSL, F: BAHD IIIa, G: BAHD IIIb. Source data are provided as a Source Data file.

Supplementary Table 1. Linear ranges, calibration curves and correlation coefficients (R^2) for escin Ia, escin Ib, protoaescigenin, esculetin, aesculin obtained by LC-QQQ-MS.

Compounds	Linear range (μg/mL)	Calibration curve	R^2
Escin Ia	9.96-1992	y=6508.4x+11.635	0.9999
Escin Ib	10.06-2012	y=5321.9x+41.251	0.9998
Protoaescigenin	0.76-152	y=76776x-32.278	0.9993
Esculetin	1.94-388	y=94504x-170.37	0.9996
Aesculin	1.78-356	y=55604x-181.53	0.9992

Supplementary Table 2. Parameters of the Multiple Reaction Monitoring (MRM) for escin Ia, escin Ib, protoaescigenin, esculetin, aesculin.

**Compounds**	**Ion mode**	**Precursor ion (*m/z*)**	**Product Ion (*m/z*)**	**Fragmentor (V)**	**Collision energy voltage (V)**
Escin Ia	positive	1131.5	969.5/789.5	170	12/15
Escin Ib	positive	1131.5	969.5/789.5	170	12/15
Esculetin	negative	339.1	176.9/163.1	90	20/30
Aesculin	negative	177.1	133.1/104.9	120	20/20
Protoaescigenin	negative	551.4	505.5/421.4	120	20/38

Supplementary Table 3. Compounds information for MALDI imaging.

Compound	Molecular formula	Adduct ion	Theoretical molecular mass	Molecular mass
Escin Ia / Escin Ib	$C_{55}H_{86}O_{24}$	$[M+K]^+$	1169.5152	1169.5088

Supplementary Table 4. Statistics of *A. chinensis* genome sequence data.

Item	Value
Total reads from Nanopore platform	3,662,658
Total bases of Nanopore reads (Gb)	34.12
N50 length of Nanopore reads (bp)	11,740
Minimum read length (bp)	1000
Maximum read length (bp)	84,436
Corrected long reads using CANU	1,229,382
Corrected bases (Gb)	18.78
N50 length of corrected reads (bp)	14,455

Supplementary Table 5. Statistics of *A. chinensis* genome assembly.

Assembly	Value
# Contigs	656
Total length (bp)	470039413
Largest contig	8581606
N50 length	2030109
GC (%)	35.55
#Polished contigs	656
Total length (bp)	470021348
Largest contig	8583990
N50 length	2049619
GC (%)	35.54
# Hi-C	
Clean Paired-end Reads	326,228,655
Unique Mapped Paired-end Reads	189,382,086
Valid Paired-end Reads	174,576,440
chr1 (bp)	31,807,429
chr2 (bp)	29,407,995
chr3 (bp)	29,233,014
chr4 (bp)	27,081,713
chr5 (bp)	26,184,402
chr6 (bp)	26,484,559
chr7 (bp)	25,384,139
chr8 (bp)	24,827,435
chr9 (bp)	23,046,765
chr10 (bp)	22,692,868
chr11 (bp)	21,891,486
chr12 (bp)	21,749,535
chr13 (bp)	21,256,607
chr14 (bp)	20,301,677
chr15 (bp)	19,447,847
chr16 (bp)	16,939,248
chr17 (bp)	17,460,947
chr18 (bp)	17,149,361
chr19 (bp)	16,763,800
chr20 (bp)	14,195,357
Total anchored (bp)	453,306,184
Unanchored (bp)	14,542,953

Supplementary Table 6. Assessment of the *A. chinensis* genome quality using Benchmarking Universal Single-Copy Orthologs (BUSCO).

**BUSCO notation**	**Number**	**Percent**
Complete BUSCOs (C)	1573	97.40%
Complete and single-copy BUSCOs (S)	1306	80.90%
Complete and duplicated BUSCOs (D)	267	16.50%
Fragmented BUSCOs (F)	18	1.10%
Missing BUSCOs (M)	23	1.50%
Total BUSCO groups searched	1614	100%

Supplementary Table 7. Annotation of *A. chinensis* TEs

Repeat Class	Elements number	Length occupied (bp)	Percentage of sequence
**Retrotransposon**	**112,183**	**125,858,360**	**26.90 %**
LINE	15,739	11,836,519	2.53 %
LTR	96,444	114,021,841	24.37 %
LTR-*Gypsy*	59,193	74,596,830	15.94 %
LTR-*Copia*	34,880	37,310,178	7.97 %
**DNA elements**	**66,062**	**27,870,276**	**5.96 %**
**Unclassified TEs**	**390,503**	**115,365,232**	**24.66 %**
Small RNA	429	182,497	0.04 %
Simple repeats	137,943	5,556,512	1.19 %
Low complexity	21,798	1,109,427	0.24 %
**Total Repeats**		**276,695,955**	59.14 %

Note: TEs, transposable elements; LTR, long terminal repeat; LINE, long interspersed nuclear elements; SINE, short interspersed nuclear elements.

Supplementary Table 8. Summary of gene annotation in *A. chinensis* genome.

Number of proteins	36603
Average gene length (bp)	2460.53
Average CDS length (bp)	1098.19
Average exons per gene	4.97
Average exon length (bp)	221.09
Average intron length (bp)	344.4
# BUSCO	
Complete BUSCOs (C)	1434 (88.80%)
Complete and single-copy BUSCOs (S)	1208 (74.80%)
Complete and duplicated BUSCOs (D)	226 (14.00%)
Fragmented BUSCOs (F)	106 (6.60%)
Missing BUSCOs (M)	74 (4.60%)
Total BUSCO groups searched	1614 (100%)

Supplementary Table 9. The K_S value for duplicated paralogous gene pairs related to terpene biosynthesis and coumarin biosynthesis in *A. chinensis* genome.

**Gene_name**	**Gene_ID**	**Gene_name**	**Gene_ID**	K_S
#Terpene biosynthesis				
DXS	AC1C306T25	DXS	AC3A21T134	0.280491
HDR	AC13A82T109	HDR	AC19A18T145	0.241994
SQS	AC13A90T97	SQS	AC19C11T29	0.2624
SQS	AC13A91T147	SQS	AC19A11T157	0.32083
MCT	AC13A199T38	MCT	AC19A148T47	0.459658
HMGR	AC13A102T193	HMGR	AC19C0T21	0.30671
MVK	AC2C14T16	MVK	AC3A41T160	0.477488
IDI	AC2A91T25	IDI	AC9C133T12	0.192534
MDS	AC3C88T16	MDS	AC5C231T33	0.207768
DXR	AC3C102T10	DXR	AC5C219T15	0.23434
HMGS	AC3A137T80	HMGS	AC5A191T108	0.18119
HMGR	AC4A263T167	HMGR	AC5A260T348	0.292022
FPS	AC5A3T158	FPS	AC8A245T219	0.183657
SQE	AC6A11T152	SQE	AC10A9T132	0.455258
HMGR	AC6A222T217	HMGR	AC14A0T85	0.27408
PMK	AC7A158T26	PMK	AC12A79T156	0.156692
ACAT	AC7A246T98	ACAT	AC12C88T31	0.247342
ACAT	AC7C41T19	ACAT	AC16C106T6	0.228154
DXS	AC7A15T198	DXS	AC16C149T14	0.37003
HDS	AC8A40T137	HDS	AC15A169T136	0.22292
MVD	AC9C189T7	MVD	AC11A73T149	0.303409
OSC	AC9C2T12	OSC	AC12A71T80	0.170068
		Average K_S		0.2758

Supplementary Table 10. Genome wide identification and expression analysis of OSC gene family members in *A. chinensis* genome.

Gene_name	Gene ID	Length (aa)	Full-length/ Partial	Flower	Pericarp	Seed	Leaf	Branch
AcOSC1	AC1C250T6	757	Full	0.12	0.00	0.30	0.08	0.25
AcOSC2	AC1C250T7	731	Full	0.00	0.00	0.00	0.00	0.00
AcOSC3	AC1A251T77	710	Full	0.03	0.03	0.00	0.00	0.00
AcOSC4	AC12A71T80	552	Partial	15.16	5.77	11.83	9.26	2.41
AcOSC5	AC20A20T18	763	Full	4.98	0.00	0.00	16.42	0.00
AcOSC6	AC15A192T141	765	Full	17.14	93.93	1116.67	27.87	14.44
AcOSC7	AC20A18T23	775	Full	0.18	0.00	0.00	0.00	0.00
AcOSC8	AC20A19T33	763	Full	0.19	0.00	0.03	4.03	0.00
AcOSC9	AC15C191T13	296	Partial	0.00	0.19	0.90	3.55	0.00
AcOSC10	AC20A22T31	159	Partial	0.00	0.00	0.00	0.00	0.00
AcOSC11	AC20C22T3	377	Partial	0.00	0.00	0.00	0.00	0.14
AcOSC12	AC20A23T36	338	Partial	0.06	0.00	0.00	0.07	0.00
AcOSC13	AC20C48T5	236	Partial	0.00	0.00	0.00	0.00	0.00
AcOSC14	AC20A49T43	761	Full	7.62	0.04	0.00	0.11	0.07
AcOSC15	AC20C49T3	763	Full	0.00	0.00	0.00	0.00	0.00
AcOSC16	AC3A166T14	218	Partial	0.00	0.00	0.00	0.00	0.00
AcOSC17	AC5C33T19	765	Full	61.02	0.00	0.75	0.00	0.00
AcOSC18	AC5A34T51	765	Full	6.31	0.09	0.87	0.00	0.00
AcOSC19	AC9C2T12	757	Full	30.15	9.81	17.75	14.11	10.73
AcOSC20	U515C0T0	369	Partial	0.00	0.00	0.00	0.00	0.00
AcOSC21	U515C0T1	369	Partial	0.00	0.00	0.00	0.00	0.00

Supplementary Table 11. The genes related to aesculin biosynthesis in *A. chinesis* genome.

Gene ID	Gene name	Leaf	Branch	Flower	Pericarp	Seed
AC11A12T142	*C4H1*	80.20	158.21	77.61	114.28	16.06
AC1P247T31	*C4H2*	4.60	0.90	17.75	9.94	11.07
AC17C169T13	*C4H3*	64.85	223.13	173.60	451.54	90.08
AC9A176T47	*4CL1*	139.96	73.65	133.34	123.66	3.76
AC20C69T2	*4CL2*	9.93	72.65	105.14	235.00	105.53
AC6A236T104	*4CL3*	4.55	6.69	6.35	59.44	12.78
AC4A229T161	*4CL4*	0.00	0.05	0.04	0.00	0.04
AC8C10T15	*F6'H1*	0.34	0.12	0.94	56.55	62.52
AC8C10T10	*F6'H2*	0.00	0.00	34.16	0.00	1.53
AC12A191T46	*F6'H3*	0.00	0.00	0.13	0.00	0.00
AC12A191T45	*F6'H4*	0.28	0.00	1.74	0.21	0.37
AC12A189T24	*F6'H5*	0.13	0.00	1.44	0.08	0.07
AC18C24T16	*PAL1*	54.73	98.10	83.95	149.65	44.36
AC18C24T18	*PAL2*	8.78	4.58	9.68	12.15	19.00
AC18C24T19	*PAL3*	0.03	0.65	19.19	8.69	0.34
AC2A266T235	*PAL4*	163.61	354.97	102.97	513.10	91.05
AC1P284T125	*PAL5*	53.71	24.08	17.11	12.13	125.55
AC15A167T55	*PAL6*	4.16	108.29	30.99	70.49	7.11
AC11A81T120	*PAL7*	30.04	46.48	53.80	244.96	48.25

Supplementary Table 12. The genes and designed primers for the functional identification of UGTs in *A. chinensis* genome. Note, AcUGTs failed to expressing protein were marked as red font.

IDs	Names	Gene IDs	Primer (F)	Primer (R)
AcUGT1		AC11P195T5.1	aaagaattcATGGAAAGAAAGCAAGAAAAG	aaagtcgacTTACAGTACATGAGTTTTCTTT
AcUGT2		AC12C34T14.1	tttGGATCCATGGGTCATCTCATCCCACT	aaaGTCGACTCAATTTTTGTGGTTCAACTTTT
AcUGT3		AC12C73T24.1	tttggatccATGGAACTATGCAAGCAAATA	tttaagcttTTAGAGTGCTTTACGGCTAA
AcUGT4		AC14C44T13.1	aaaggatccATGGCTAGCTTTGGTCATAT	tttaagcttTTACTCGTTTTTGTAATTTTGAA
AcUGT5		AC15C184T29.1	aaaggatccATGAAGAAAGCAGAGCTGAT	aaagtcgacTTATGGAAAATTCATACCAATG
AcUGT6		AC15C85T3.1	aaaggatccATGTCACGAGGACTTGTCC	aaagtcgacCATTGTTGTTTTCTTGTCCCTT
AcUGT7		AC8P181T12.1	tttaagcttTTATGTGCTTGTTTCATGATAT	aaaGTCGACTTGATAGCCTGTCTTTTAGCT
AcUGT8		AC18C29T8.1	aaagaattcATGGCCGCCACCCAAATCCA	aaactgcagTTAATAGTGCTTGCTCTGCC
AcUGT9	**AcUGT84A56**	AC19C29T9.1	aaaggatccATGGGTTCTCAATCTCTTGT	aaactgcagCTAGCAAGTGACCTCCAC
AcUGT10		AC19C4T23.1	tttGTCGACATGGCTATGGCTGAACCAGA	tttAAGCTTTCATGGTTTAGTACTTGCTG
AcUGT11		AC1C283T16.1	aaaggatccATGTTTCCATGGCTAGCTTA	tttgtcgacTCAAAGAGACCGAGTTGAA
AcUGT12		AC2C229T6.1	aaaggatccATGCAGAAAGCAGAGCTTG	tttgtcgacTTATGAAATGTTGTCGATCAC
AcUGT13		AC2C230T2.1	aaaggatccATGCAGAAAGCGGAGCTT	tttgtcgacTTATGAAATGTTGTCGATCA
AcUGT14		AC10C43T8.1	aaaggatccATGAGTTGAAAGTAGTTGGTT	aaactgcagAGAGAGCTTGTAAGATAAAAC
AcUGT15		AC4A198T170.1	tttGGATCCATGGCGGATGAACAAGTATTG	tttAAGCTTTTACTCGCTGCAAGGATGAT
AcUGT16		AC4P194T62.1	aaaggatccATGGGTCATCTCATCCCAC	tttaagcttTCAATTTTTGTTGTTCCACTT
AcUGT17	**AcUGT92G7**	**AC7C90T5.1**	tttggatccATGGCTTGCAGCAGTGATG	tttaagcttATAAGTGATTTGCAGGACGT
AcUGT18		AC8C14T21.1	aaaggaaggatttcaCTAAACTCCTTATCAAGATAAA	aaagtcgacTCAACGGCAGTGATTCTGG
AcUGT19		AC9P140T38.1	aaagaattcATGGTTTCAGAAGATGATCAA	aaactgcagTCTGATATCCTTTATTAGATTC

Supplementary Table 13. Kinetic assay of AcUGT92G7 and AcUGT84A56 towards esculetin.

AcUGTs	Substrate	V_{max}(nmol·min^{-1})	K_M(μM)	k_{cat}(s^{-1})	k_{cat}/K_M(s^{-1}·M^{-1})
AcUGT92G7	Esculetin	0.029±0.003	48.957±6.324	0.010±0.001	210.653±25.579
AcUGT84A56	Esculetin	0.005±0.000	177.250±3.748	0.001±0.000	7.050±0.107

Supplementary Table 14. The designed primers for the functional identification of escin Ia biosynthetic genes in *A. chinensis* genome.

Names	Gene IDs	Primers (F)	Primers (R)
AcOSC6	AC15A192T141.1	ATGTGGAAACTGAAGACAGCAGA	CTAAGCAGCAGTAATCTTGGATGG
AcCYP716A278	AC15C190T21.1	ATGGCTTGCAGCAGTGATG	ATAAGTGATTTGCAGGACGT
AcCYP716A275	AC8C1T11.1	ATGGAGTTGTTCTTGGTTATACTC	TCAGTGATGAGGAATAAGACGAA
AcBAHD1	AC15C192T11.1	tttGAATTCATGGAAGTCCGCATCATTTCTA	aaaGTCGACTCATATCACATTCACACTAGGA
AcBAHD3	AC15P192T121.1	tttGAATTCATGGAAGTCCGCATCATTTCT	tttGTCGACTCATATCACATTCACACTAGGA
AcBAHD5	AC15P193T53.1	tttGAATTCATGGATGTCAAAATCATTTCTAC	tttGTCGACTTAGGCTGGATTGAAAGAAGC
AcBAHD6	AC6E162T1	ATGGGTCGCGGATCCGAATTCATGGAAAAAAGCAGCATCATTTCT	TGCGGCCGCAAGCTTGTCGACTGTGGTTGGATTAAAGGAAGCA
AcCSL1	AC8C1T8.1	tttGGATCCGATGGGTGATCTTCATTCCC	tttGTCGACTTAATTTGTTTTGCTCTTGCTT

Supplementary Table 15. The designed primers for the functional identification of aesculin biosynthetic genes in *A. chinensis* genome.

Names	Gene IDs	Primers (F)	Primers (R)
Ac4CL1	AC6A236T104.1	gccatggctgatatcggatccATGGCGGACAAAGAAGATCATC	tgcggccgcaagcttgtcgacTTAATTGGGTAGACCGGTGGC
Ac4CL2	AC9A176T47.1	gccatggctgatatcggatccATGATTTCCATTGCTGATCATCC	tgcggccgcaagcttgtcgacTTAAGAAATTGGGGAGGGTGG
Ac4CL3	AC20C69T2.1	gccatggctgatatcggatccATGTCTCCCCAATTAAGCCAAG	tgcggccgcaagcttgtcgacTTAGTTTGGATATCCAGCTTCTAGCT
AcF6'H1	AC8C10T10.1	gccatggctgatatcggatccATGGCACCAGCAATCTCTATCTC	tgcggccgcaagcttgtcgacTTAGTCGGCGATCTTTGCAAA
AcF6'H2	AC8C10T15.1	gccatggctgatatcggatccATGGCACCAGCGATCTCTATCT	tgcggccgcaagcttgtcgacTCAAACTTCTGTGATCTTTGCAAAA
AcF6'H3	AC10A213T138.1	gccatggctgatatcggatccATGGCTCCAAGCTTGGAAGAA	tgcggccgcaagcttgtcgacTTAATTACTAACATGAGCGAAATTAAGAGA
AcF6'H4	AC3A54T95.1	gccatggctgatatcggatccATGTCTCCAGGCTTGGAGGAA	tgcggccgcaagcttgtcgacTCATGGTCGATTAACATGAGCG

Supplementary Table 16. Specific parameters of LC-MS/MS analysis for identification of enzymatic products.

Detection targets	Column	Mobile phases	Gradient programs	Mass spectrometer type/ scan type/ polarity
Enzymatic assays of AcCSL1	Agilent Infinity Lab Poroshell 120 EC-C18 column (1.9 µm, 2.1×50mm)	A, acetonitrile;<br>B, water containing 0.1% acetic acid	0 min, 95% B; 7 min, 5% B; 8-11 min, 95% B	LC-QTOF-MS/MS;<br>Auto MS/MS mode;<br>Negative mode
Enzymatic assays of AcBAHDs with protoaescigenin being substrate	Waters Acquity UPLC® BEH C18 column (1.7 µm, 100 × 2.1 mm)	A, water containing 0.1% formic acid;<br>B, acetonitrile	0 min, 10%B<br>10-12min, 95%B;<br>13-16min, 10%B	LC-QQQ-MS/MS;<br>MS2 scan mode;<br>Negative mode
Analysis of purified 22-*O*-acetylprotoaescigenin	Waters Acquity UPLC® BEH C18 column (1.7 µm, 100 × 2.1 mm)	A, water containing 0.1% formic acid;<br>B, acetonitrile	0 min, 10%B<br>10-12min, 95%B;<br>13-16min, 10%B	LC-QTOF-MS/MS;<br>Auto MS/MS mode;<br>Negative mode.
Enzymatic assays of AcBAHDs with desacetylescin Ia being substrate	Waters Acquity UPLC® BEH C18 column (1.7 µm, 100 × 2.1 mm)	A, water containing 0.1% formic acid;<br>B, acetonitrile	0-4 min, 40%B;<br>10-12 min, 100%B;<br>12.1-15 min, 40%B	LC-QTOF-MS/MS;<br>Auto MS/MS mode;<br>Negative mode
Enzymatic assays of AcF6Hs	Agilent Eclipse Plus C18 column (RRHD 1.8µm, 2.1×50mm)	A, water containing 0.1% formic acid;<br>B, acetonitrile	0-2min, 5%B;<br>2-13min, 6%B;<br>13-13.1min, 95%B;<br>13.1-14.1min, 95%B;<br>14.1-14.5min, 5%B	LC-QTOF-MS/MS<br>Auto MS/MS mode;<br>Negative mode
Enzymatic assays of AcUGTs	Agilent Eclipse Plus C18 column (RRHD 1.8µm, 2.1×50mm)	A, acetonitrile; B, water containing 0.1% acetic acid	0 min, 95%B;<br>5 min, 50%B;<br>6-9 min, 95%B	LC-QTOF-MS/MS<br>Auto MS/MS mode;<br>Positive mode

Supplementary Table 17. Plasmids and strains and used in this study.

Plasmids	Description	Source
pZE12-luc	$P_L$lacO1, *colE* ori, luc, Amp^r, high copy	This study
pCS27	$P_L$lacO1, P15A ori, Kan^r, medium copy	This study
pCS-TPTA-HpaBC	pCS27, PLlacO1-TPTA and PLlacO1-HpaBC	1
pCS-TAL	pCS27, *Rg*TAL from *Rhodotorula glutinis*	2
pZE-649T	pZE12-luc, *AcF6'H1*, *Ac4CL2*, *AcUGT92G7* and *RgTAL* encoding genes	This study

Strains	Description	Source
DH5α	F^-ψ80d*Zlac*△ (*Zlac*YA-*arg*F) U169 *end*A1 *rec*A1 *hsd*R17 (r_k^-, m_k^+) *sup*E44λ-*thi*-1 *gyr*A96 *rel*A1 *pho*A	TransGen Biotech
BW25113	rrnBT14 ΔZlacWJ16 hsdR514 ΔaraBADAH33 ΔrhaBADLD78	CGSC
BWΔ*pykA*Δ*pykF*	BW25113 deleting genes pykA and pykF	This study
BW1	BWΔ*pykA*Δ*pykF*, pCS-TPTA-HpaBC and pZE-649T	This study

Supplementary Table 18. Primers used in construction for heterologous expression.

Primers	Sequence (5'-3')
KpnI-F6H-F	GGGAAAGGTACCatggctccaacactcttgacaacccaattc
SphI-F6H-R	GGGAAAGCATGCttagatcttggcgtaatcgacggttttc
SphI-4cl-F	GGGAAAGCATGCaggagatataccatggcgccacaagaacaagcagtttctc
NotI-4cl-R	GGGAAAGCGGCCGCttacaatccatttgctagttttgccctcagatc
BamHI-RBS-92-F	GGGAAAGGATCCaggagatataccATGGCTTGCAGCAGTGATGATGAG
BamHI-92-R	GGGAAAGGATCCGCGGCCGCTTAAGTGATTTGCAGGACGTGATTG
NotI-RBS-TAL-F	GGGAAAGCGGCCGCaggagatataccATGGCGCCTCGCCCGACTTCGCAAAG
XhoI-TAL-R	GGGAAACTCGAGTTATGCCAGCATCTTCAGCAGAACATTGTTG

[a] Underlines denote restriction sites.

Supplementary references

1. Li, X. *et al.* Establishing an artificial pathway for efficient biosynthesis of hydroxytyrosol. *ACS Synth. Biol.* **7**, 647–654 (2018).
2. Chen, Z., Sun, X., Li, Y., Yan, Y. & Yuan, Q. Metabolic engineering of *Escherichia coli* for microbial synthesis of monolignols. *Metab. Eng.* **39**, 102–109 (2017).
