# Supplementary material for: Characterization of the horse chestnut genome reveals the evolution of aescin and aesculin biosynthesis
Source: Nat Commun. 2023 Oct 13;14:6470. doi: 10.1038/s41467-023-42253-y (PMC10576086; doi:10.1038/s41467-023-42253-y)
Supplement: Supplementary file 3 — Description of Additional Supplementary Files [file 41467_2023_42253_MOESM3_ESM.pdf]

Description of Additional Supplementary Files

File Name: Supplementary Data 1
Description: The functional annotation and GO enrichment analyses of the retained genes from the recent WGD event of A. chinensis genome.

File Name: Supplementary Data 2
Description: Genome wide identification and expression analysis of CYP450 gene family members in A. chinensis genome.

File Name: Supplementary Data 3
Description: Genome wide identification and expression analysis of BAHD gene family members in A. chinensis genome.

File Name: Supplementary Data 4
Description: Genome wide identification and expression analysis of UGT gene family members in A. chinensis genome.

File Name: Supplementary Data 5
Description: Predicted gene clusters related to triterpenoid biosynthesis in A. chinensis genome and their gene expression patterns in different tissues.

File Name: Supplementary Data 6
Description: The expression and annotation for the co-expression genes, which clustered into "turquoise" module of WGCNA.
