# Supplementary material for: Characterization of the horse chestnut genome reveals the evolution of aescin and aesculin biosynthesis
Source: Nat Commun. 2023 Oct 13;14:6470. doi: 10.1038/s41467-023-42253-y (PMC10576086; doi:10.1038/s41467-023-42253-y)
Supplement: Supplementary file 10 — Reporting Summary [file 41467_2023_42253_MOESM10_ESM.pdf]

nature portfolio

Corresponding author(s): Zhichao Xu, Xinxiao Sun, Shilin Chen

Last updated by author(s): Sep 24, 2023

Reporting Summary

Nature Portfolio wishes to improve the reproducibility of the work that we publish. This form provides structure for consistency and transparency in reporting. For further information on Nature Portfolio policies, see our Editorial Policies and the Editorial Policy Checklist.

Statistics

For all statistical analyses, confirm that the following items are present in the figure legend, table legend, main text, or Methods section.

n/a	Confirmed	
☐	☒	The exact sample size (*n*) for each experimental group/condition, given as a discrete number and unit of measurement
☐	☒	A statement on whether measurements were taken from distinct samples or whether the same sample was measured repeatedly
☐	☒	The statistical test(s) used AND whether they are one- or two-sided<br>*Only common tests should be described solely by name; describe more complex techniques in the Methods section.*
☒	☐	A description of all covariates tested
☐	☒	A description of any assumptions or corrections, such as tests of normality and adjustment for multiple comparisons
☐	☒	A full description of the statistical parameters including central tendency (e.g. means) or other basic estimates (e.g. regression coefficient) AND variation (e.g. standard deviation) or associated estimates of uncertainty (e.g. confidence intervals)
☐	☒	For null hypothesis testing, the test statistic (e.g. *F*, *t*, *r*) with confidence intervals, effect sizes, degrees of freedom and *P* value noted<br>*Give P values as exact values whenever suitable.*
☒	☐	For Bayesian analysis, information on the choice of priors and Markov chain Monte Carlo settings
☒	☐	For hierarchical and complex designs, identification of the appropriate level for tests and full reporting of outcomes
☒	☐	Estimates of effect sizes (e.g. Cohen's *d*, Pearson's *r*), indicating how they were calculated

Our web collection on statistics for biologists contains articles on many of the points above.

Software and code

Policy information about availability of computer code

Data collection	Zeiss Axio M2 microscope (Zeiss, Germany); MALDI system (TransMIT GmbH, Giessen, Germany); Q Exactive HF Orbitrap mass spectrometer (Thermo Fisher Scientific, Bremen, Germany); SMALDIControl software package (TransMIT GmbH, Giessen, Germany); 6470 Triple Quadrupole mass spectrometer (Agilent Technologies, Santa Clara, CA, USA); 1290 UHPLC system (Agilent Technologies, Santa Clara, CA, USA); Agilent Eclipse Plus C18 column (RRHD 1.8µm, 2.1×50mm); MassHunter software (Agilent Technologies, Santa Clara, CA, USA); ONT GridION X5 platform (v.9.4.1; Oxford Nanopore Technologies); NextSeq 500 platform; Agilent Technology 7890 GC, coupled with a 7000C Triple Quadrupole MS (Agilent Technologies, Santa Clara, CA USA); Bruker Avance Neo 600 MHz Magnetic Resonance Spectrometer;
Data analysis	Jellyfish (v.2.0); Guppy (v.1.8.5); CANU (v.1.5); SMARTdenovo; Pilon (v.1.24); BUSCO (v4); BWA-MEM (v.0.7.17); 3D-DNA; HISAT2 (v.2.0.5); FeatureCounts (v.1.6.3); Weighted Gene Co-expression Network Analysis (WGCNA, R package); RepeatModeler (v.1.0.9); Trinity (v.2.2.0); TransDecoder (v.2.1.0); MAKER (v.2.31.9); RaxML (v.8.2.10); PAML (v.4.9); CAFE (v.2.1); MCscan (Python version); plantiSMASH online pipeline (http://plantismash.secondarymetabolites.org/). The specific-parameters used in this study have been described in the section of "methods" in detail. All scripts and software are available from Github as indicated in the methods.

For manuscripts utilizing custom algorithms or software that are central to the research but not yet described in published literature, software must be made available to editors and reviewers. We strongly encourage code deposition in a community repository (e.g. GitHub). See the Nature Portfolio guidelines for submitting code & software for further information.

Data

Policy information about availability of data

All manuscripts must include a data availability statement. This statement should provide the following information, where applicable:

- Accession codes, unique identifiers, or web links for publicly available datasets
- A description of any restrictions on data availability
- For clinical datasets or third party data, please ensure that the statement adheres to our policy

The raw data of genome and transcriptome sequencing generated in this study have been deposited in the Genome Sequence Archive in BIG Data Center, Beijing Institute of Genomics (BIG), Chinese Academy of Sciences, under accession code CRA009101 (https://ngdc.cncb.ac.cn/gsa/browse/CRA009101) and CRA009093 (https://ngdc.cncb.ac.cn/gsa/browse/CRA009093) that are publicly accessible at http://bigd.big.ac.cn/gsa. The assembled genome and gene structures of A. chinensis have been deposited in Figshare (https://doi.org/10.6084/m9.figshare.21350865). Source data are provided with this paper.

Research involving human participants, their data, or biological material

Policy information about studies with human participants or human data. See also policy information about sex, gender (identity/presentation), and sexual orientation and race, ethnicity and racism.

Reporting on sex and gender	not applicable
Reporting on race, ethnicity, or other socially relevant groupings	not applicable
Population characteristics	not applicable
Recruitment	not applicable
Ethics oversight	not applicable

Note that full information on the approval of the study protocol must also be provided in the manuscript.

Field-specific reporting

Please select the one below that is the best fit for your research. If you are not sure, read the appropriate sections before making your selection.

☒ Life sciences ☐ Behavioural & social sciences ☐ Ecological, evolutionary & environmental sciences

For a reference copy of the document with all sections, see nature.com/documents/nr-reporting-summary-flat.pdf

Life sciences study design

All studies must disclose on these points even when the disclosure is negative.

Sample size	For genome sequencing, amount of young leaves (more than 10 g) from an individual Aesculus chinensis plant were collected. For RNA-Seq, two to four independent biological samples were used. For LC/MS-MS analysis of different tissues, three independent biological samples for each tissue were collected to valid statistical analyses.
Data exclusions	For genome and transcriptome analysis, the low-quality sequencing reads were excluded. For phylogenetic analysis, the short protein-coding sequences were excluded.
Replication	For RNA-Seq, two to four independent biological replicates were performed, and for metabolome analysis, three independent biological replicates were performed. All attempts at replication were successful.
Randomization	For genome sequencing, an individual strong Aesculus chinensis plant with no random sampling was collected. For RNA-Seq and LC/MS-MS analysis, the samples were allocated into experimental groups at random.
Blinding	Blinding is not applicable in our study because it does not involve subjects which receive different treatments. All experiments were done by analyzing data derived from different biological replicates directly.

Reporting for specific materials, systems and methods

We require information from authors about some types of materials, experimental systems and methods used in many studies. Here, indicate whether each material, system or method listed is relevant to your study. If you are not sure if a list item applies to your research, read the appropriate section before selecting a response.

Materials & experimental systems

n/a	Involved in the study
☒	☐ Antibodies
☒	☐ Eukaryotic cell lines
☒	☐ Palaeontology and archaeology
☒	☐ Animals and other organisms
☒	☐ Clinical data
☒	☐ Dual use research of concern
☐	☒ Plants

Methods

n/a	Involved in the study
☒	☐ ChIP-seq
☐	☒ Flow cytometry
☒	☐ MRI-based neuroimaging

Flow Cytometry

Plots

Confirm that:

☒ The axis labels state the marker and fluorochrome used (e.g. CD4-FITC).

☒ The axis scales are clearly visible. Include numbers along axes only for bottom left plot of group (a 'group' is an analysis of identical markers).

☒ All plots are contour plots with outliers or pseudocolor plots.

☒ A numerical value for number of cells or percentage (with statistics) is provided.

Methodology

Sample preparation	Nuclei from young leaves were isolated and darkly stained by PI for 15 min. The stained nuclei were resuspended in the 1% PBS buffer.
Instrument	BD Accuri C6
Software	BD Accuri C6
Cell population abundance	Abundance >10000 cells were respectively collected from the young leaves from referenced samples and tested samples.
Gating strategy	Total nuclei populations were gated using relative fluorescence intensity.

☒ Tick this box to confirm that a figure exemplifying the gating strategy is provided in the Supplementary Information.
